# State of the Art in Actuation of Micro/Nanorobots for Biomedical Applications

**DOI:** 10.1002/smsc.202300211

**Published:** 2024-02-02

**Authors:** Ahmed Elnaggar, Seungyeop Kang, Mingzhen Tian, Bing Han, Meysam Keshavarz

**Affiliations:** ^1^ Mechanical Engineering Imperial College London Bessemer Building South Kensington Campus Exhibition Road Kensington London SW7 2AZ UK; ^2^ The Hamlyn Centre for Robotic Surgery Imperial College London Bessemer Building South Kensington Campus Exhibition Road Kensington London SW7 2AZ UK; ^3^ Institute of Medical Robotics School of Biomedical Engineering Shanghai Jiao Tong University Dongchuan Road Shanghai 200240 China; ^4^ Imperial College London Electrical and Electronic Engineering Bessemer Building South Kensington Campus Exhibition Road Kensington London SW7 2AZ UK

**Keywords:** electrical actuation, magnetic actuation, micro/nanorobots, optical actuation, ultrasonic actuation

## Abstract

The emergence of micro/nanorobotics stands poised to revolutionize various biomedical applications, given its potential to offer precision, reduced invasiveness, and enhanced functionality. In the face of such potential, understanding the mechanisms that drive these tiny robots, especially their actuation techniques, becomes critical. Although there is a surge in research dedicated to micro/nanorobotics, there exists a gap in consolidating the diverse actuation strategies and their suitability for biomedical applications. This comprehensive review seeks to bridge this gap by providing an in‐depth evaluation of the current actuation techniques employed by micro/nanorobots, particularly emphasizing their relevance and potential for clinical translation. The discussion starts by elucidating the different actuation strategies, ranging from magnetic, electric, acoustic, light‐based, to chemical and biological mechanisms. Then, various examples and meticulous assessment of each technique are offered, spotlighting their respective merits and limitations within a biomedical context. This review illuminates the transformative capabilities of these actuation methods in medicine. It not only highlights the progress made in this burgeoning field but also underscores the areas that require further exploration and development.

## Graphical abstract

Micro/nanorobotics has unlocked possibilities in diverse fields, including minimally invasive surgery, regenerative medicine, and targeted drug delivery. This comprehensive review article highlights the remarkable progress in this rapidly growing field, offering a thorough evaluation of the current state of the art in actuation techniques utilized by micro/nanorobots, with a specific emphasis on their biomedical applications.

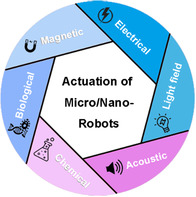

## 1 Introduction

Micro/nanorobots have experienced a long and evolving history, with its roots tracing back to the proposal of physicist Richard Feynman in the early 1960s to manipulate individual atoms and molecules. Initially, micro/nanorobots were designed for biomedical applications such as drug delivery and cell manipulation, with the goal of providing minimally invasive and targeted therapies. Since then, the field has broadened to encompass a wide range of applications including tissue engineering, biosensing, and surgical procedures. The small size of these robots is crucial for their use in biomedical engineering and healthcare, allowing them to access and interact with biological systems at the micro‐ and nanoscales.^[^ ^1^ ^]^ In medical imaging, their small size enables them to navigate through narrow blood vessels to a maximum of 13 μm error throughout their journey or tissues to provide detailed images at the cellular level.^[^ ^2^, ^3^ ^]^ In tissue engineering, they can manipulate cells and tissues at a small scale to create complex structures and promote tissue growth and regeneration.^[^ ^4^, ^5^ ^]^ Additionally, the small size of micro/nanorobots reduces the risk of harm to patients through minimizing invasiveness and can also be designed to be biocompatible to reduce the risk of rejection by the immune system. However, it was not until the advent of nanotechnology, material science, and bioengineering that the field began to take shape.

**Figure 1:**
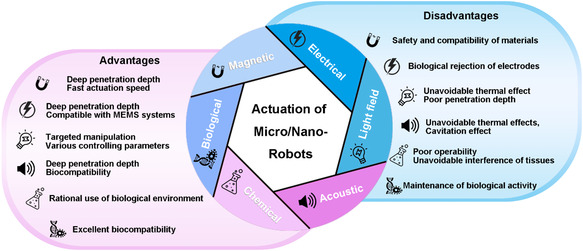
Schematic illustration of various actuation techniques actuation.

The terms “microrobots” and “nanorobots” are frequently used interchangeably and the knowledge presented here applies to both types of robots.^[^ ^6^, ^7^, ^8^ ^]^ For the purposes of this review, robots with body length below 1 mm are discussed. From 1 μm to 1 mm, the robot will be considered a microrobot and below 1 μm will be considered a nanorobot. Considering that movement of tiny object in fluids is governed by low Reynolds numbers, the inertial force is negligible compared to the viscous force, micro/nano‐robots must be fully operational with little‐to‐no supervision, highly efficient, easily controlled, and inexpensive in mass manufacturing.^[^ ^7^, ^9^, ^10^ ^]^ They also need to be large enough to handle information from various sensory systems and small enough to enter the body without physically degrading live tissues.^[^ ^11^, ^12^ ^]^ As a result, actuation requires a continuous power supply, which presents a challenge due to the difficulty of loading micro/nanorobots with power sources such as batteries and engines due to their small size. The major focus of research in the field of micro/nanorobots is actuation technology (shown in **Figure** **1**), and a combination of actuation methods will be necessary to utilize the benefits while avoiding the drawbacks. The advantages and drawbacks of various actuation methods are briefly discussed in **Table** **1**, followed by a comprehensive evaluation in the respective section.

**Table 1:** Comparison of actuation methods for micro/nanorobots

Actuation method	Advantages	Disadvantages
Magnetic field	Powerful penetrating power, no harm to living biological systems, fast actuation speed	Unavoidable safety problems when applying high intensity magnetic fields over 8 T
Electric field	Adjustable electric field intensity, powerful penetrating power	Imitations on biomedical applications of electrodes
Light field	Precise targeting, various controlling parameters, such as power density, wavelength, pulse frequency, polarities and so on	Irreversible harm to biological materials, poor light transmission
Acoustic field	Adjustable for different heights, biocompatible within a certain frequency range, powerful penetrating power, and actuation force	Prolonged actuation time may cause thermal effects, low imaging, and cavitation effect
Chemical	Larger and faster spread	Lack of feedback fuel safety issues, nontoxic urea, biocompatibility
Biological	Good biocompatibility, cause little rejection reaction	Need to maintain activity, limited range, need ultra‐clean operating environment

**Figure 2:**
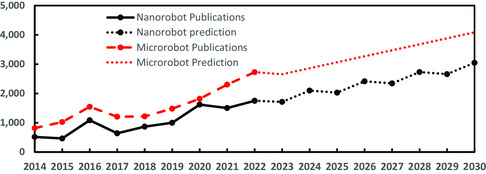
A summary of publications related to micro/nanorobotics from 2014, with predictions up to 2030. Source: Dimensions AI. Keywords: Microrobot and Nanorobot respectively. Microrobot and nanorobot publications are set to surpass 3500 and 2500 publications respectively before 2030.

In recent years, the development of micro/nanorobots has advanced rapidly.^[^ ^13^ ^]^ As predicted in **Figure** **2**, the number of publications in the field of microrobotics and nanorobotics is expected to surpass 3000 and 2500 respectively, with a particular emphasis on intelligent materials, actuation techniques, and control systems. Publications for nanorobots are less, as they are relatively harder to fabricate, navigate, control, and actuate smaller robots with lower Reynolds numbers as discussed above. Hence, swarm nanorobots have emerged as a promising new field, due to their easier control and higher efficacy in payload delivery.^[^ ^14^, ^15^ ^]^ Sophisticated and efficient micro/nanorobots are carried out successively, making them much more feasible for biomedical applications. Undoubtedly, the application of microrobots is mutually reinforcing in terms of materials, manufacturing, actuation, and certain application scenarios. Materials must exhibit a high biocompatibility to ensure interactions with biological systems do not elicit adverse reactions. Advances in manufacturing techniques, particularly in micro and nanofabrication, make the design and production of microrobots more precise and feasible. Micro and nanofabrication methods enable the realization of small‐scale and complex structures suitable for navigating within biological organisms. Excellent reviews, focusing on one of these topics, have been carried out recently, such as the processing methods,^[^ ^16^, ^17^, ^18^, ^19^ ^]^ active materials, and^[^ ^20^ ^]^ manipulation methods.^[^ ^21^, ^22^, ^23^, ^24^ ^]^

The biomedical application of micro/nanorobots is a highly specialized interdisciplinary field that requires the collaborative efforts of multiple experts to continue advancing this technology and achieving meaningful biological applications.

In this review article, we delve into the interdisciplinary realm of micro/nanorobots, with a primary focus on actuation and their biomedical applications. We evaluate and compare the major actuation techniques, including magnetic, electric, acoustic, light, chemical, biological, and hybrid methods, emphasizing their respective advantages and limitations. This review provides a comprehensive overview of the latest advancements in the use of micro/nanorobots for a diverse range of biomedical applications. Our aim is to shed light on the future trajectory of this field and to identify areas primed for further research and innovation. Our study investigates the criteria for selecting different actuation methods, aligning them with pertinent biomedical applications and their inherent challenges. Additionally, we explore the myriad solutions proposed by researchers to tackle diseases and complications in various regions of the human body.

## 2 Magnetic Actuation

Over time, the medical usage of magnets expanded to include modalities such as the application of magnets to the body and the peroral administration of magnetite powder in drugs.^[^ ^21^, ^25^, ^26^, ^27^ ^]^ The understanding of magnetism and its application in medicine has evolved significantly, leading to the development of magnetic diagnostic techniques and treatment modalities.^[^ ^28^, ^29^, ^30^ ^]^ Notably, in 1973, Paul Lauterbur pioneered magnetic resonance imaging (MRI), a medical imaging technology that revolutionized the detection of various conditions, including tumours, brain damage, and spinal injuries. Today, magnets are extensively used in the medical field for a wide range of purposes, including MRI, transcranial magnetic stimulation, magnetic therapy, drug delivery, dentistry, and hyperthermia treatments for cancer.^[^ ^31^, ^32^, ^33^, ^34^ ^]^ The historical perspective and evolution of magnetic technologies for medical applications continue to be an area of active research and development.^[^ ^29^ ^]^

Magnetic actuation is a well‐studied method and commonly used method for controlling the movement of micro and nanorobots due to its noninvasiveness and biocompatibility. Magnetic fields can be used to apply forces on magnetic particles embedded in the robot, which can be used to propel the robot or manipulate its position. One of the key challenges in magnetic actuation is the design of the magnetic fields and the magnetic particles to achieve precise control over the movement of robots.

There are two types of magnetic actuation mechanisms: time varying, which include rotating, oscillating, and stepping magnetic fields, and field gradient (also known as inhomogeneous). Magnetic field gradients were frequently used to manage magnetic micro/nanoparticles (NPs) in earlier magnetic micro/nanorobot designs. Today, one of the most often applied time‐varying fields is rotating magnetic fields. They are frequently employed to actuate helical robots, one of the most popular types of microrobots, which are propelled by the induction of rolling,^[^ ^35^ ^]^ corkscrew,^[^ ^36^ ^]^ and spin‐top^[^ ^37^ ^]^ motions. Previous studies have also revealed that rotating magnetic fields can also control magnetic particle aggregation and several other intriguing formations.^[^ ^38^, ^39^, ^40^ ^]^ On the other hand, flexible robots are usually the focal point of oscillating magnetic fields which are discussed more thoroughly in Section 2.2. Magnetic fields with stepping patterns have ON/OFF phases and are periodic magnetic fields. They can create a power stroke that causes the magnetic cilium of a robot to move in a wave‐like manner.^[^ ^41^ ^]^

One example of the advancement of magnetically actuated microrobots due to MRI comes in a study conducted by Go et al.^[^ ^42^ ^]^ They created microrobots that are capable of precision navigation to tumor‐feeding channels for transcatheter liver chemoembolization in vivo and these can be seen in real time by X‐ray and MRI. As shown in real‐time X‐ray imaging, these microrobots offer targeted administration of medicinal and imaging agents, guided by magnetic forces from the actuation module. They are made with a special composition of hydrogel‐enveloped porous structure and magnetic nanoparticles (MNPs). The benefit of postoperative MRI tracking guarantees thorough monitoring, and the microrobots gradually deteriorate over time, reducing long‐term damage. This method promises a step toward sophisticated, minimally invasive medical procedures, promising increased accuracy and efficacy in cancer therapy.

Targeted cell delivery has been an emerging topic for magnetically actuated microrobots. The creation of a degradable magnet‐driven and image‐guided microrobot for the precise administration of modified stem cells in the treatment of orthotopic liver tumours is described in a study by Wei et al.^[^ ^43^ ^]^ A composite material was used to create this cutting‐edge microrobot, which has a burr‐like porous sphere structure and balances mechanical strength, degradability, and magnetic actuation capabilities. These microrobots include engineered stem cells that are finely enclosed and can be precisely discharged due to the optimized microrobot structure. A gradient magnetic field directs the motion of the microrobot, and special photoacoustic imaging technology precisely directs its path. In preliminary studies on nude mice, the microrobots showed outstanding efficiency in suppressing tumor growth by precisely delivering therapeutic cells when administered via the portal vein.

Using a magnetically driven microrobot with a porous shape to distribute targeted cells is a promising method to improve the poor targeting performance of the mesenchymal stem cell (MSC) in tissue regeneration. Go et al.^[^ ^44^ ^]^ developed a medical microrobot system based on human adipose‐derived MSCs for knee cartilage regeneration and presented an in vivo trial to confirm the effectiveness of the microrobot using a cartilage lesion model. A microrobot body that can support MSCs, an electromagnetic actuation mechanism for 3D targeting of the microrobot, and a magnet to fix the microrobot to the injured cartilage make up the microrobot system. Each part was created and developed with clinical safety, patient accessibility, and staff accessibility in mind. The effectiveness of the microrobot system was then evaluated in a rabbit knee cartilage defect model in order to acquire approval for a clinical trial.

Other studies have also made progress in the field of microrobots for cell‐based therapy. Using a magnetic gradient field‐driven mechanism, Li et al.^[^ ^45^ ^]^ described a microrobot with a burr‐like porous spherical shape for carrying and delivering specific cells in vivo. The construction involved 3D laser lithography, and Ni and Ti coatings were applied for magnetic actuation and biocompatibility, respectively. The proposed microrobot design might improve magnetic driving capability, encourage cell‐carrying capacity, and benefit cell viability, according to numerical and experimental tests. In vivo transport of cell‐cultured microrobots in zebrafish embryos proved that microrobots laden with cells can be automatically directed to reach a specified spot utilizing a self‐built electromagnetic coil system. In vitro tests revealed that cells from the microrobot were either immediately discharged onto the desired spot or were able to transit through the blood vessel‐like microchannel to reach the delivery area. The transported cells might be spontaneously released from the microrobot to the surrounding tissues. On nude mice, additional in vivo cell‐releasing experiments were carried out, followed by a histological analysis. This study offers a microrobotic device platform for cell‐based therapy and regenerative medicine.

Moreover, for 3D cultivation and precise transport of stem cells in vitro, ex vivo, and in vivo, magnetic microrobots were created by Jeon et al.^[^ ^46^ ^]^ Microrobot‐attached hippocampal neural stem cells multiplied and differentiated into astrocytes, oligodendrocytes, and neurons. Additionally, colorectal cancer cells were moved by microrobots to a network of in vitro liver‐tumor micro‐organs in a body on a chip that contained tumor microtissue. Additionally, a mouse brain slice and a rat brain blood artery were used to control the microrobots. Finally, a intraperitoneal cavity of a nude mouse was used to handle microrobots carrying MSCs generated from the nose. The outcomes show that microrobots have a future in the delivery and cultivation of stem cells.

The immune system, which serves as a natural defense mechanism against outside dangers, is one issue that magnetic microbots for medical interventions must encounter. Therefore, stealth zwitterionic microrobots that are nonimmunogenic and can evade immune cell detection are presented in a study by Cabanach et al.^[^ ^47^ ^]^ For two‐photon polymerization (2PP) 3D microprinting, the researchers have created entirely zwitterionic photoresists, enabling the development of hydrogel microrobots with a variety of capabilities. These microrobots are capable of magnetic actuation, biomolecule encapsulation, and surface functionalization for drug administration, in addition to having variable mechanical properties, antibiofouling traits, and nonimmunogenic traits. A remarkable accomplishment in the field of microrobotics is the ability of these stealth microrobots to avoid identification by macrophage cells of the innate immune system after thorough examination spanning more than 90 h. In addition to removing a major barrier to the development of biocompatible microrobots, this adaptable zwitterionic material also acts as a fundamental toolkit for nonimmunogenic materials, revolutionizing medical microrobotic technologies and opening new opportunities in bioengineering and biomedical applications.

### 2.1 Magnetic Materials

Fabricated microrobots have been endowed with magnetic properties by applying a tin coating using elements such as nickel (Ni) or cobalt (Co), along with a layer of titanium (Ti) or gold (Au) to enhance biocompatibility.^[^ ^34^, ^46^, ^48^ ^]^ However, this approach presents a limitation to their translational application in biomedical settings, as these materials are nonresorbable and may lead to long‐term complications if they persist in the body. Consequently, the utilization of MNPs in the fabrication of micro/nanorobots has garnered considerable interest. Hence, it is crucial to examine the commonly employed magnetic materials for the production of micro/nanorobots, as they serve as a prerequisite for achieving magnetic responsiveness.

Magnetic components are commonly used to control micro/nano‐objects in nanostructures. The magnetic susceptibility (*x*_m_) is a key parameter that categorizes magnetic materials into four types: ferromagnetic, ferrimagnetic, paramagnetic, and diamagnetic, depending on whether *x*_m_ is greater than or equal to zero.

Paramagnetic materials have weak attraction to magnetic fields, and their magnetic moments are randomly oriented. Ferromagnetic materials exhibit a potent magnetic field due to their great attraction to magnetic fields and the alignment of their magnetic moments. Ferrimagnetic materials, even if their magnetic moments are not aligned, are nonetheless magnetic. Comparatively to ferromagnetic materials, ferrimagnetic materials show a less total magnetic field. Diamagnetic materials are weakly repelled by a magnetic field because their magnetic moments are arbitrarily oriented in opposition to one another; they cancel one another out. Ferromagnetic and ferrimagnetic materials are below the Curie temperature, while materials above the Curie temperature are paramagnetic. Magnetic fields only weakly attract or repel paramagnetic and diamagnetic materials, respectively. Additionally, these materials are unable to retain any magnetization once the magnetic field is withdrawn. Ferro‐ and ferrimagnetic materials are strongly attracted to magnetic fields, and they exhibit leftover magnetization, also known as remanence. Usually, high remanence is a feature of hard‐ferromagnetic materials, which are also known as permanent magnets. Soft ferromagnets, in contrast, exhibit low remanence. Both soft and hard magnets exhibit a hysteretic behavior, which means that a coercive magnetic field is required to demagnetize these materials. This coercivity is large for hard magnets and small for soft magnets. Superparamagnets, on the other hand, are a special class of materials in which features of both ferromagnets and paramagnets converge such as high susceptibility, no remanence, and no coercivity.^[^ ^49^, ^50^, ^51^, ^52^ ^]^ **Table** **2** provides more in‐depth information on diverse types of magnetic materials. Although there are some examples of nanorobots made from paramagnetic and diamagnetic materials, most small‐scale magnetic robots are constructed using ferromagnetic and superparamagnetic compounds.

**Table 2:** Magnetic materials for nanorobots with their respective properties listed

	Diamagnetic	Paramagnetic	Ferromagnetic	Ferrimagnetic
Strength of Magnetism	Weak Magnetism	Weak Magnetism	Strong Magnetism	Strong Magnetism
Behaviour Under Non‐Uniform Field	Tend to move from high to low region	Tend to move from low to high region	Tend to move from low to high region	Tend to move from low to high region
Permeability	Little less than unity	Little greater than unity	Very high	Very high
Susceptibility	Little less than unity and negative	Little greater than unity and positive	Very high and positive	Very high and positive
Effect of Temperature	No Effect	With the rise of temperature, it becomes a diamagnetic	Above curie point it becomes a paramagnetic	Above curie point it becomes a paramagnetic
Advantages	Compared with permanent magnets, there no friction and 3‐D control	Compared with ferromagnetic particles, there is no magnetization and coercivity	Minimal power consumption, room temperature adaptability, self‐stability, easy to miniaturize	Low toxicity
Materials	Gold, silver, copper, bismuth, carbon (diamond) Zinc, Antimony	Aluminium, platinum, titanium	Iron, nickel, cobalt	Magnetite (Fe_3_O_4_), garnet, yttrium iron garnet (YIG)
References	[184, 185, 186]	[184, 185, 186]	[187, 188, 189]	[190]

Equation (1) shows that a small‐scale magnetic robot with a volume v will exhibit magnetization **M** when subjected to an external magnetic field **B**. The apparatus will experience an attractive force (or a repulsive force if it is a diamagnet) when exposed to a magnetic field gradient **ΔB**. To reduce its energy, the magnetic robot will align itself so that its easy magnetization axis (also known as easy axis) is parallel to the direction of the applied magnetic field. Equation (2) represents the torque, **T**, involved in this alignment.^[^ ^22^ ^]^ The easy magnetization axis is often determined by the shape of the material (shape anisotropy), but it can also be controlled by certain crystal orientations (crystalline anisotropy).^[^ ^53^ ^]^ Additionally, it is possible to program the easy magnetization axis, for example, by aligning magnetic nanostructures with a matrix of a composite component or by premagnetizing a material in a certain direction.

(1) F=v(M × ∇)B

(2) T=vM×B

### 2.2 Oscillating Electromagnetic Actuation

**Figure 3:**
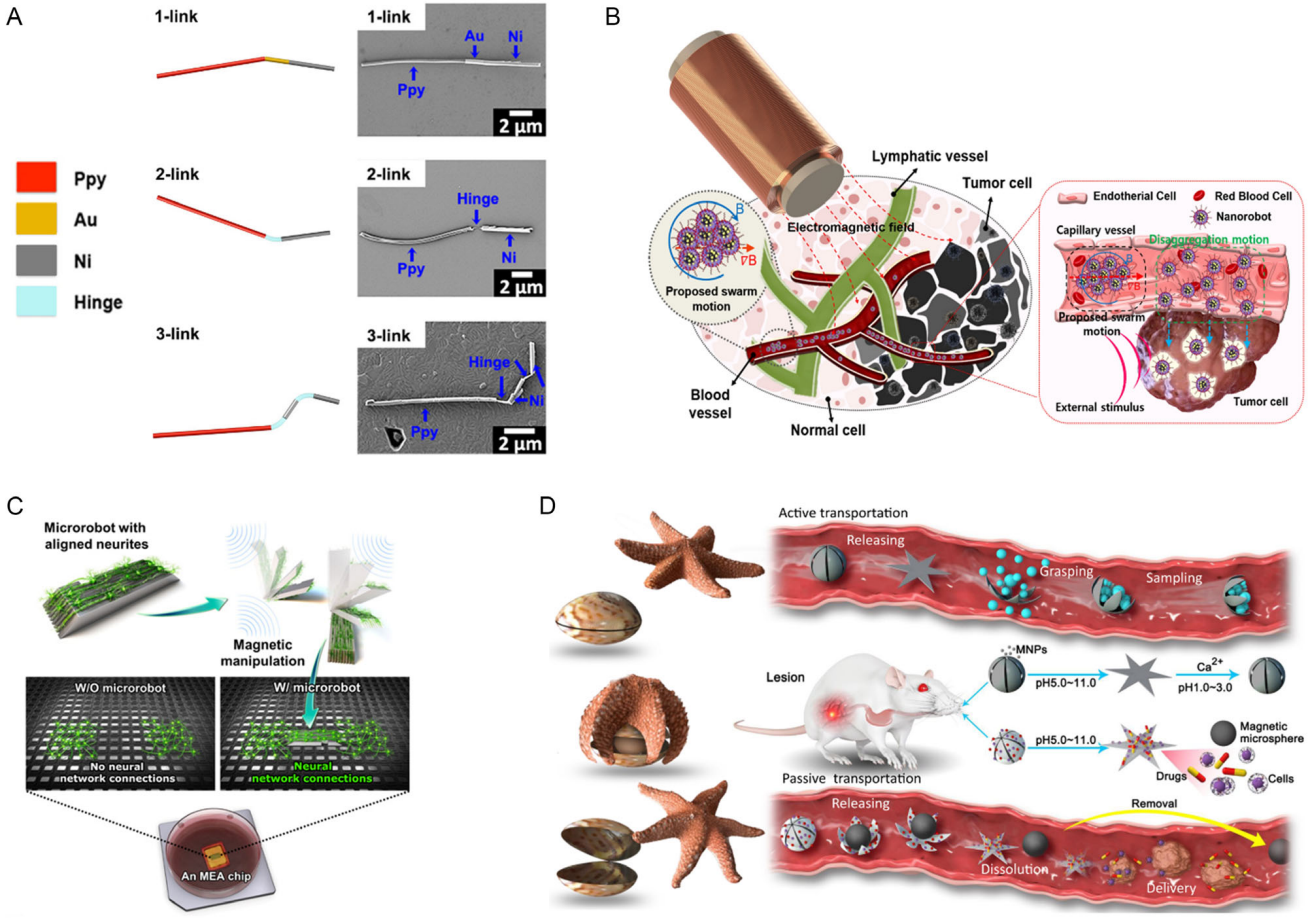
A) Schematic of one‐, two‐, and three‐link swimmers with the corresponding scanning electron microscopy (SEM) images. Each swimmer is around 15.5 μm long overall. Reproduced with permission.^[^ ^51^ ^]^ Copyright 2015, American Chemical Society. B) Conceptual schematic of targeted drug delivery to manipulate nanorobots to targeted area by Ennead Electromagnets Actuation System with proposed swarm motion. Reproduced under the terms of the CC‐BY Creative Commons Attribution 4.0 International license (https://creativecommons.org/licenses/by/4.0).^[^ ^54^ ^]^ Copyright 2021, The Authors, published by Springer Nature. C) Schematic illustration of a magnetically actuated microrobot for neural networks based on the active construction between two neural clusters utilizing the microrobot on a high‐density multielectrode array chip. Reproduced with permission.^[^ ^61^ ^]^ Copyright 2020, The Authors, published by AAAS. From ref. [61]. Copyright, The Authors, some rights reserved; exclusive licensee AAAS. Distributed under a CC BY‐NC 4.0 license http://creativecommons.org/licenses/by-nc/4.0/. Reprinted with permission from AAAS. D) Schematic of an ionic SMMR end effector (ISME) in the GI tract. For the active transportation, the ISME conducts target sampling under enteric pH or ionic stimulus after releasing a payload. For the passive transportation, the ISME releases a payload and dissolves for efficient delivery of drugs or cells to the intestines under pH 5–11. Reproduced under the terms of the CC‐BY Creative Commons Attribution 4.0 International license (https://creativecommons.org/licenses/by/4.0).^[^ ^64^ ^]^ Copyright 2021, The Authors, published by Springer Nature.

**Figure 4:**
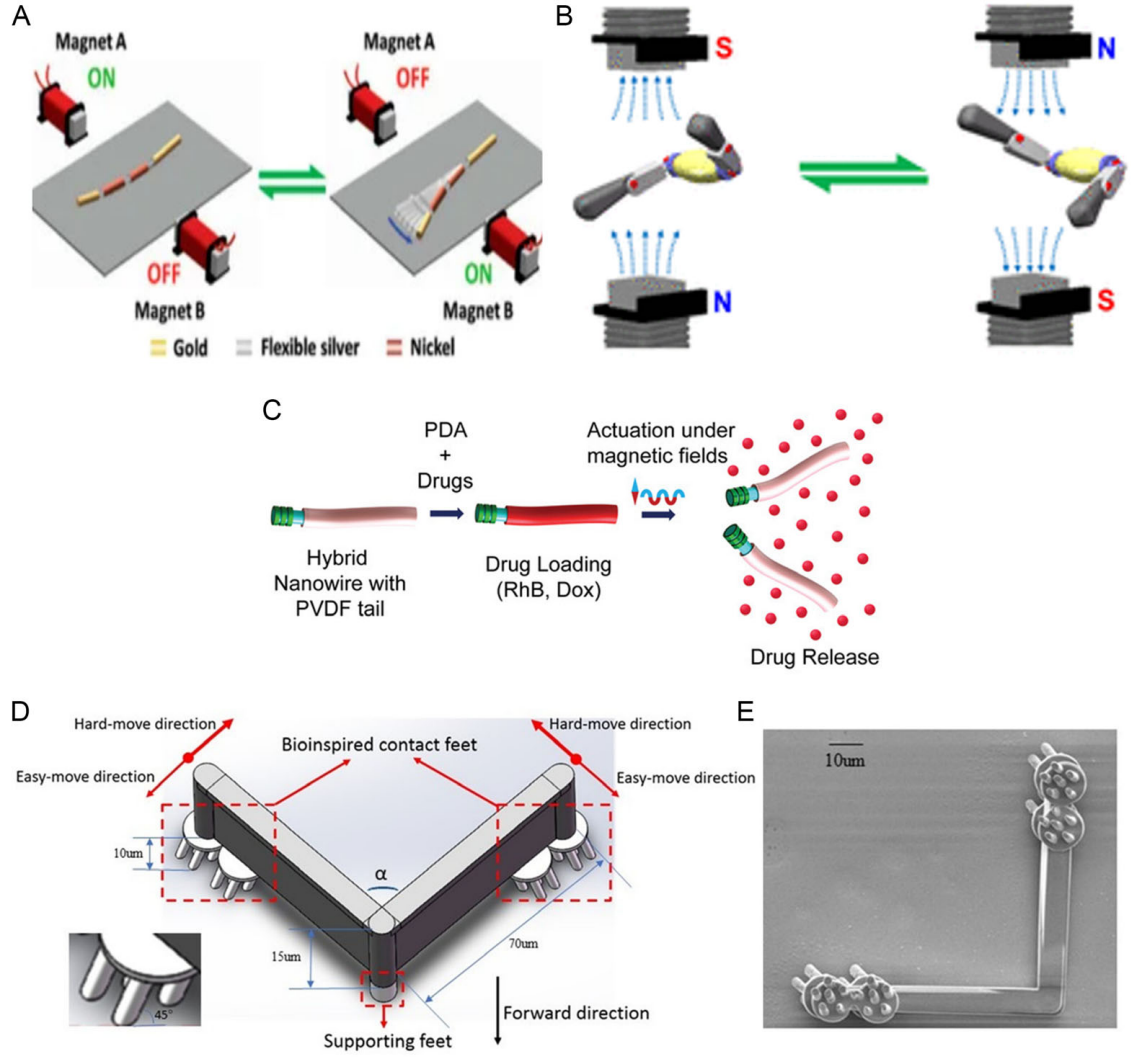
A) Magnetic propulsion of an artificial nanofish using a planar oscillating magnetic field. Biocompatible magnetic microrobots are made in a single step. Reproduced with permission.^[^ ^55^ ^]^ Copyright 2016, Wiley‐VCH. B) Schematic for two‐arm freestyle propulsion of nanoswimmer in an oscillating magnetic field with setup. Reproduced with permission.^[^ ^57^ ^]^ Copyright 2017, American Chemical Society. C) Depiction of hybrid nanoeels with polydopamine and drugs, subsequently accompanied by magnetically driven drug release. Reproduced with permission.^[^ ^58^ ^]^ Copyright 2019, Wiley‐VCH. D) Diagram of the structure of designed microwalker. E) SEM image of designed microwalker. D,E) Reproduced under the terms of the CC‐BY Creative Commons Attribution 4.0 International license (https://creativecommons.org/licenses/by/4.0).^[^ ^60^ ^]^ Copyright 2022, The Authors, published by Wiley‐VCH.

An oscillating electromagnetic field refers to a rapidly changing electromagnetic field that alternates in polarity or direction over time. In the realm of micro/nanorobotics, this phenomenon finds valuable applications. By harnessing oscillating electromagnetic fields, micro/nanorobots can be manipulated and controlled with precision. These fields can be used to induce controlled motion in these tiny robotic devices, allowing them to navigate through complex environments or perform targeted tasks. For micro/nanorobots, there is a growing demand for nanoscale navigation and control precision, as well as for nanorobots capable of achieving nanoscale accuracy in manipulation. These requirements are essential for a wide range of applications, such as targeted drug delivery, noninvasive medical procedures, and the manipulation of small‐scale structures (shown in **Figure** **3**B).^[^ ^54^ ^]^ Inspired by the swimming processes of fish or bacterial flagellum, researchers have proposed wagging propulsion as a promising mode of propulsion. In an oscillating magnetic field, a nanoscale magnetic component tends to change its state and follow changes in the field strength, allowing for effective wagging propulsion. This approach has been successfully used in many experiments (shown in **Figure** **4**A) to power nanomotors, such as transporting red blood cells (RBCs) using a magnetic filament.^[^ ^55^ ^]^ A magnetic field consisting of a homogenous static field **B**_***x***_ = B_*x*_ *x* and a sinusoidal field **B**_***y***_ = B_y_sin(2*π*ft)y was used to achieve wagging propulsion.^[^ ^43^ ^]^ Transverse field including the filament tail would swing as it oscillated, propelling the nanorobots along **B**_***x***_.^[^ ^46^ ^]^ A magnetically actuated robotic system (shown in Figure 4B) capable of completely automated manipulation of cells and nanobeads was created by Steager et al. utilizing a U‐shaped magnetic robot that was actuated by a gradient magnetic field produced by five electromagnetic coil controllers. To fabricate the U‐shaped 30 × 30 × 10 μm^3^ microtransporter, a mixture of MNPs and photoresist was cured by a photolithography technique to produce the U‐shaped pattern.^[^ ^56^ ^]^ Under the control of a gradient magnetic field, the robot could catch and autonomously distribute nanobeads infused with chemicals to particular regions in neurons, which has potential implications in the delivery of tailored medications.

Furthermore, Jang et al. reported on the first wire multilink nanorobots (Figure 3A) that were pushed by two groups of coplanar, opposing coil pairs that were offset by 90°.^[^ ^51^ ^]^ These two sets of coils produced two sinusoidal oscillating fields (*B*_*x*_ =*B*_A_cos(*θ*sin(2*πft*)) and *B*_*y*_ = *B*_A_sin(*θ*sin(2*πft*)), which they used to impose a planar oscillating magnetic field on the nanorobots. The overall lengths of the swimmers are ≈15.5 μm with diameters of 200 nm. They found that the nanoswimmers, which had different numbers of connections, exhibited dissimilar swimming and wagging states. Additionally, the three‐link swimmers, which had an S‐like motion of their tails, could travel faster and more effectively than the one‐ and two‐link swimmers. This feature is attributed to the number of linkages, which denotes a greater degree of freedom, enabling them to move more freely and achieve better propulsion.

Similarly, Li et al. created a fish‐like magnetic actuation nanorobot (diameter 200 nm; length 4.8 μm, shown in Figure 4A) with a passive gold segment acting as the head, two active nickel segments acting as the body, and a passive gold segment acting as the caudal fin.^[^ ^55^ ^]^ This three‐segmented fish‐like nanorobot is connected by a flexible structure made of porous silver. The swimming mode of robot, which is influenced by an oscillating magnetic field on a plane, is strikingly similar to that of a fish propulsion. The robot can therefore travel at relatively high speeds and demonstrated an acceptable propulsion efficiency. The oscillating magnetic field with a frequency of 11 Hz achieved a maximum speed of 30.9 μm s^−1^, which is equivalent to a dimensionless speed of 0.6 body lengths per revolution. The effective propulsion characteristic of this nanofish has significant potential for a variety of future practical applications, including nanoscale assembly, manipulation, and nanomedicine.

A new type of magnetically actuated nanorobots (shown in Figure 4B) with a freestyle swimming mode was developed which consists of a central gold body segment, two nickel arm segments, and flexible porous silver hinges that enable symmetric swinging motion.^[^ ^57^ ^]^ By magnetizing the nickel arm segments and applying an oscillating magnetic field, the robot can achieve a freestyle swimming mode with the arms swinging in various directions. The magnetic field can be changed remotely to change speed and orientation the robot. The top speed is 59.6 μm s^−1^ or almost 12 body lengths per second relative to the object. This was one of the early examples of a nonplanar swinging swimming mode which was produced using oscillating magnetic fields. The freestyle swimmer nanorobot outperformed the fish‐like nanorobot in terms of maximal movement speed and propulsion efficiency. This innovative swimming mechanism and its enticing performance bring up new design opportunities for remotely controlled nanorobots used in nanoscale biomedical applications.

The development of the “nano‐eel” hybrid nanorobot (shown in Figure 4C). by Mushtaq et al. represents a significant advancement in nanotechnology.^[^ ^58^ ^]^ By mimicking the movement of an electric eel, this soft nanorobot demonstrates the potential for intelligent and flexible devices capable of executing complex tasks. The use of organic polymers such as polypyrrole and polyvinylidene fluoride copolymer in the construction of the nano‐eel highlights the importance of biomimicry in nanotechnology. These materials allow for the creation of devices that can interact with biological systems in a noninvasive and effective manner. The nickel ring attached to the head of the nano‐eel enables magnetic actuation. The ferroelectric tail bends and its electric polarization changes when an alternating magnetic field is applied. By taking advantage of the magnetically coupled piezoelectric effect, an on‐demand release of therapeutic cargo loaded on the surface of the piezoelectric tail has been demonstrated, and this release has been electrostatically enhanced. Furthermore, a pulsatile release of payloads, which could be utilized for drug delivery, has been shown by this approach.

A novel method that draws inspiration from fish biomechanics has been presented by Wang et al.^[^ ^59^ ^]^ outlining a magnetic microrobot designed specifically for the effective transportation of microparticles along liquid surfaces. This 850 μm × 500 μm × 200 μm microrobot is cleverly designed as a monolithic sheet structure with inherent magnetic properties, in contrast to its aquatic counterparts that are propelled by flexible caudal fins. This was accomplished by carefully incorporating magnetic particles into polydimethylsiloxane during the meticulous fabrication process. The intentional thickness variation in different portions of the fish‐shaped microrobot enhances its mobility. This design cleverly leverages the differences in surrounding liquid levels when subjected to an oscillating magnetic field. Through meticulous theoretical analysis and computer simulations, the fundamental propulsion mechanism has been methodically disassembled. Validation and refinement have then been achieved through methodical experimental assessments. Particularly striking is the achievement of a maximum transport speed of 1.2 mm s^−1^, equivalent to three times the diameter of the microball per second. More applications in the field of micromanipulation are anticipated for the suggested microrobot and transportation technique.

Moreover, a magnetically driven and frictional anisotropy‐based microwalker that can be used in an in vivo, nonliquid environment is proposed by Jia et al.^[^ ^60^ ^]^ As shown in Figure 4D,E, the microwalker is made of two stiff pieces that are each 70 mm long and joined together rigidly. Tentacles that resemble parallel gecko setae are positioned at the base of the segments to act as contact feet and create friction with the contact surface. The microwalker is completely constructed using 2PP and 3D laser lithography from biocompatible materials. The microwalker has the ability to move on surfaces in liquid and nonliquid environments unlike previous microswimmers which can only move in liquid environments. Additionally, it can ascend a slope that is only influenced by a planar magnetic field. The microwalker can move on surfaces in nonliquid environments in addition to moving in a liquid environment like current microswimmers. Additionally, it can ascend a slope that is only influenced by a planar magnetic field.

This, in turn, causes the magnetic head module nickel–polypyrrole to oscillate, and due to the piezoelectric effect as described in Equation (3) and (4), the electric polarization changes, causing the ferroelectric tail to bend in the opposite direction, creating a wave‐like motion similar to that of an eel.

Equation (3) and (4) describes the piezoelectric effect.

(3) $$D_{m}=d_{\text{mi }}\sigma_{i}+\varepsilon_{\text{mi}}E_{k}$$

(4) D∝Q

Experiments indicate that the strength and frequency of magnetic fields can alter the motion of nano‐eel, ranging from surface walking to 3D swinging and spiraling. In addition, the nano‐eel can be directed precisely to the target site for medication release using the appropriate magnetic fields. The nano‐eel has substantially progressed the field of drug delivery. In their research, drug delivery to cancer cells was accomplished with a magnetic field of 10 mT and 7 Hz. In the drug release mode, cancer cell death is 35% efficient, compared to 10% in the swimming mode. The capacity to use nanorobots for targeted drug delivery was quantitatively confirmed. This will support the usage of nanorobots in clinical settings and lay the groundwork for further study.

Another exciting area of medical application for magnetically actuated nanorobots is the development of active neural networks and the targeted delivery to nerve cells (shown in Figure 3C).^[^ ^61^ ^]^ This advances the study of neural networks and neural connectivity with reproducibility, selectivity, and precise connection. This offers a potential foundation for innovative, programmable artificial neural network models in vitro as well as other possible additions of artificial intelligence models in the field. The reason for neural network model development is because the nanorobots must be capable of reacting in real time, in accordance with different changes and requirements that come from the surrounding dynamic environment.^[^ ^62^ ^]^

Currently, the study of swarm behavior of micro/nanorobots is a rapidly growing area of research which is especially one of the strengths of magnetic actuation. As researchers continue to advance their understanding of single‐magnetic field‐driven nanorobots, they are also interested in exploring how groups of nanorobots can function together to perform tasks that are difficult or impossible for a single robot. To explore the behavior of groups of nanorobots, Hsu et al. created a control platform with a scalable system and a modular circuit architecture and up to 16 nanorobots were coordinated to perform minimally invasive, collision‐free 2D position control.^[^ ^63^ ^]^ According to experiments, the maximal degree of freedom can reach 288 and random jitter can be introduced with a 100% success rate. Swarm control will therefore likely become even more popular issue in nanorobot research.

Zheng et al. developed a sea‐star‐like microrobot with flexible tentacles that can efficiently conform to the exterior contours of any target with autonomous deformation in a liquid environment for gripping and releasing (shown in Figure 3D).^[^ ^64^ ^]^ Ranging between 200 and 600 μm in size, they were created by electrodepositing alginate hydrogel. Inspiration for this project came from the movement process of starfish preying on shellfish. The robot can be activated in a variety of ways, such as by encasing MNPs or magnetic nanospheres in the body of microrobots, as shown in **Table** **3**. After arriving at the desired location, several functions such as attaching, delivering, and sampling are carried out by autonomous deformation and posture adjustment. An illustrative case involves the use of biodegradable biomaterials to facilitate self‐deformation of microrobots based on environmental cues, effectively addressing prior challenges related to the seamless operation of microrobots within confined living environments. Therefore, a growing number of researchers are becoming interested in its use in soft robotics, minimally invasive medical devices, and smart materials.

**Table 3:** Comparison of magnetic microswimmers

Nanorobot	Illustrationa)	Body length [μm]	*U*_max_ [μm s^−1^]	*U*_max_ (Body Length [s])	References
Magnetic multilink microswimmers	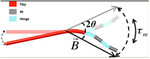	15.5	14.4	0.9	[51]
Fish‐like microswimmers	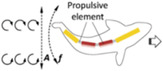	4.8	30.9	6.9	[55]
Freestyle magnetic microswimmers	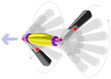	4.8	59.6	12	[57]

a) Illustration for magentic multilink microswimmers: Reproduced with permission.^[^ ^51^ ^]^ Copyright 2015, American Chemical Society. Illustration for fish‐like microswimmers: Reproduced with permission.^[^ ^55^ ^]^ Copyright 2016, Wiley‐VCH. Illustration for freestyle magnetic microswimmers: Reproduced with permission.^[^ ^57^ ^]^ Copyright 2017, American Chemical Society.

### 2.3 Electromagnetic Devices

Electromagnetic devices play a crucial role in the actuation of microrobots, enabling their precise control and manipulation for various applications. These devices utilize the interaction between magnetic fields and the microrobots to achieve motion and functionality. Diverse types of electromagnetic actuation systems have been developed and studied. One common approach involves the use of rotating magnetic fields to manipulate magnetic microrobots within a workspace.^[^ ^65^, ^66^ ^]^ Magnetic microrobotics holds promise for improving minimally invasive surgery, offering benefits such as limited incisions, reduced bleeding and postoperative pain, and faster recovery time.^[^ ^67^ ^]^ Fabrication techniques such as soft lithography and 3D structuring have been employed to create electromagnetic actuators for microrobots.^[^ ^68^ ^]^ The precise control of microrobots through controlled magnetic fields is an area of significant interest, particularly in the context of soft microrobots that can navigate controllably under the influence of magnetic fields.^[^ ^69^ ^]^ Furthermore, researchers have explored the design and control methods of microrobots in microfluidic systems using electromagnetic fields, enabling both linear and rotational motion of the microrobots.^[^ ^70^ ^]^ Magnetic actuation, utilizing low‐frequency and quasistatic magnetic fields, is commonly employed to propel magnetically responsive microrobots and apply forces and torques.^[^ ^71^ ^]^ Electromagnetic microactuators have been developed, including unidirectional and bidirectional configurations, utilizing magnetic composites for various applications.^[^ ^72^ ^]^

**Figure 5:**
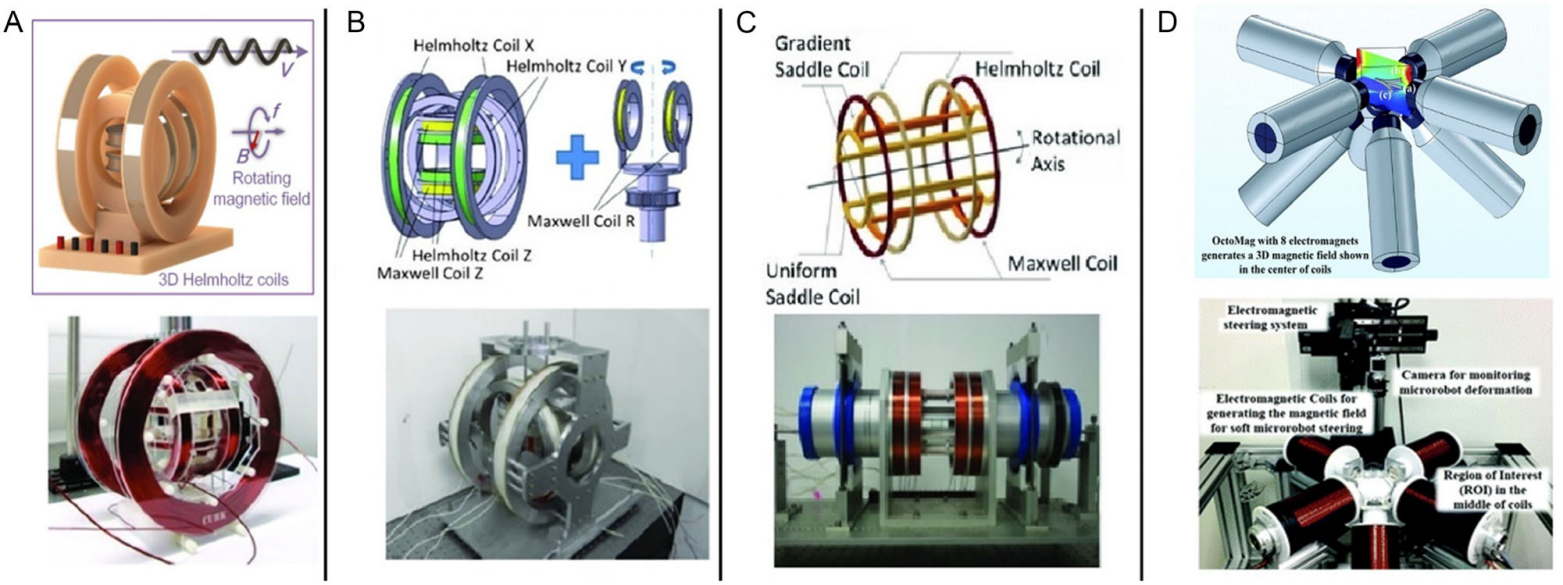
Experimental setups for magnetically driven micro/nanorobots and various magnetic actuation systems. A) Electromagnetic actuation system using triaxial circular Helmholtz coils. Top: Reproduced with permission.^[^ ^25^ ^]^ Copyright 2022, Elsevier. Bottom: Reproduced under the terms of the CC‐BY Creative Commons Attribution 4.0 International license (https://creativecommons.org/licenses/by/4.0).^[^ ^21^ ^]^ Copyright 2020, The Authors, published by Wiley‐VCH. B) Electromagnetic actuation system using a system consisting of a triaxial Helmholtz coil and two Maxwell coils. Reproduced with permission.^[^ ^178^ ^]^ Copyright 2010, Elsevier. C) Electromagnetic actuation system with a Helmholtz coil, Maxwell coil, uniform saddle coil, and gradient saddle coil. Reproduced with permission.^[^ ^179^ ^]^ Copyright 2010, Elsevier. D) The OctoMag microrobot steering actuation system. Reproduced under the terms of the CC‐BY Creative Commons Attribution 4.0 International license (https://creativecommons.org/licenses/by/4.0).^[^ ^180^ ^]^ Copyright 2019, The Authors, published by Mary Ann Liebert.

Magnetic fields are produced in electromagnet‐based magnetic actuation devices by passing currents through coils. A ferromagnetic core, which has the ability to concentrate and amplify the magnetic field and field gradient, is wrapped in insulated copper wires to create a standard electromagnet. To prevent the effects of hysteresis, the core is frequently made of an ideal soft magnetic material. The desired configuration of magnetic fields, such as spinning fields, oscillating fields, alternating fields, and conical fields, can be achieved by on‐demand setting of current in each coil. Specialized electromagnet systems are made up of various coil configurations, such as the Helmholtz coil, Maxwell coil, saddle coil, and double‐saddle Golay coil. First and foremost is the most important arrangement, the Helmholtz coil, which consists of two circular, coaxial coils with equal radii and identical current flow directions. Such a magnetic actuation system is suitable for magnetic torque control since the field produced by the Helmholtz coil is uniform at the middle of the coils. Two pairs of Helmholtz coils or a triaxial Helmholtz coil can produce arbitrary homogeneous magnetic fields in a 2D plane or 3D space, respectively. The most typical mechanism for controlling magnetic small‐scale robots is a triaxial circular Helmholtz coil (**Figure** **5**A). Helmholtz coils and other types of coils can be combined to create systems with multi‐degrees of freedom (DOF) capability. The Maxwell coil is made up of two equal‐radius circular coaxial coils; however, the current flowing through each coil is the opposite. Uniform magnetic field gradients can be produced using Maxwell coils, saddle coils can produce either a uniform or a gradient field, and double‐saddle Golay coils can provide a transverse gradient. Through the management of both magnetic forces and torques, a magnetic small‐scale robot is capable of 3D locomotion using a magnetic manipulation system with a triaxial Helmholtz coil and two Maxwell coils. (Figure 5B). The updated design, which uses four different coil pairs—a Helmholtz coil, a Maxwell coil, a rotatory uniform saddle coil, and a rotatory gradient saddle coil—takes up less space and uses less driving power (Figure 5C). Saddle coil and Golay coil with tubular structure are preferred due to their excellent space efficiency and ability to accommodate the human body in actual clinical applications of biomedical micro/nanorobots. For instance, a common MRI scanner in clinical practice uses two orthogonal Golay coils and a Maxwell coil.^[^ ^22^, ^73^ ^]^

For Figure 5D, a more advanced steering system is available in the OctoMag. The one shown in the figure can create a 3D magnetic field with a maximum intensity of 40 mT OctoMags produced by eight electromagnets. There are 5° of freedom: two rotational and three translational. The special capabilities of OctoMag are a result of its use of complicated nonuniform magnetic fields, which takes advantage of a linear representation of the coupled field contributions of several soft‐magnetic‐core electromagnets functioning cooperatively. OctoMag has potential benefits in other medical applications as well as micromanipulation under an optical microscope. The system can be used with visual tracking to offer exact positioning under closed‐loop control, but it can also be used without it to provide visual input to the human operator during direct teleoperation. OctoMag was created to operate intraocular microrobots for minimally invasive retinal therapy and diagnosis, but it can also function as a wireless micromanipulator system when used with a light microscope. Researchers Kummer et al. at ETH Zurich created the OctoMag and were able to wirelessly puncture CAM blood arteries of a chicken embryo in vitro as a proof‐of‐concept experiment.^[^ ^74^ ^]^ A more compact version of the OctoMag was discovered in the MiniMag in 2014. It has been reported that OctoMag and MiniMag can be used to remotely control micro/nanorobots for stem cell therapy in rat brains,^[^ ^46^ ^]^ minimally invasive ocular surgery,^[^ ^75^ ^]^ and targeted drug delivery.^[^ ^55^ ^]^

The OctoMag and the MiniMag are examples of a stationary electromagnetic control system, one of the three magnetic control systems, with the two other types being permanent magnet control system and mobile electromagnet control. Stationary electromagnetic coils are used by the stationary electromagnet control system to regulate field intensity and turn on and off the external magnetic field. Through a variety of current inputs, this system provides flexibility in controlling and has the ability to modify the magnetic field. However, while having a weaker magnetic field than core electromagnets, hollow‐core electromagnets are easier to model and regulate. The magnetic field produced by a single coil is computed independently and superimposed using a linear method; however, several coils can decouple the overall magnetic field. There are drawbacks to the electromagnetic coil, such as higher temperature from Joule heating and the requirement for cooling systems. Additionally, poor flexibility and challenges may result from the intense magnetic field needed for large‐scale operation. The input of current may change with an increase in system volume, necessitating a revision of the input calculation models. The input connection changes from linear to nonlinear as the cored electromagnet hits saturation, which has an impact on the modeling and control of coil.^[^ ^21^ ^]^ Researchers suggested an improved stationary electromagnetic control device in 2018.^[^ ^76^ ^]^ The BatMag system, which has 6° of freedom, can autonomously operate the same and different microrobots in 3D. BatMag naturally boosts to 6‐DOF control in comparison to OctoMag and MiniMag. Researchers create a strong magnetic field and a magnetic field gradient using many electromagnetic coils, which can simulate the gravitational interaction force. The final design for BatMag consists of nine fixed electromagnetic coils, each with a permanent magnet within. A thermal management technique was developed that transfers heat from the coil to the aluminum radiator utilizing a water‐cooling system. Researchers have created and validated relevant algorithms that the microrobot can use in the interim. The BatMag system can independently manage various and identical microrobots while keeping flexibility and excellent controllability by taking advantage of the inhomogeneity of the magnetic field that is generated. Additionally, it is thought to offer enormous promise for managing microrobot clusters or therapeutically suitable imaging applications.^[^ ^77^ ^]^

Systems for controlling permanent magnets provide a magnetic field without external power, which lowers the amount of heat generated in the workspace. There are restrictions to the flexibility of permanent magnet systems, however, due to the constraints imposed by the degrees of freedom the robotic arm joint.^[^ ^21^ ^]^ Higher strength and gradient strength are produced as a result, improving the flexibility and efficiency of the robot. The magnetic field strength, however, is continuous, necessitating precise design and sophisticated algorithms. Additionally, because permanent magnets cannot turn off the field source, they present a danger to patients and medical staff due to magnetic field attenuation.^[^ ^78^ ^]^ In 2017, Ryan et al. carried out a new magnetic control system. This device controls the microrobot precisely and remotely in five degrees of freedom using eight revolving permanent magnets.^[^ ^79^ ^]^ Additionally, it can strengthen the gradient and magnetic field while producing the least amount of heat. For rotating movement without moving parts, the system simply has to manage the external permanent magnets, which can be safer and easier. Eight permanent magnets make up the system, and each one has an independent rotational motion that can be used to direct the movement of the microrobot in 3D. It is possible to limit the amount of heat generated in the workspace by positioning the motor away from the magnet. Moreover, Ryan and Diller suggested that to address the drawbacks of the earlier permanent magnet systems, the angular positions of each magnet be adjusted until the magnetic field and magnetic field gradient are both 0 which is comparable to shutting off the external magnetic field. The gradient descent approach is used by the system to determine the corresponding local minimum value for feedback control after weighing the differences between the magnetic field and the force measurement unit. Even in situations where working space is constrained or positions are absolutely forbidden, this driving system with eight revolving permanent magnets can achieve a high level of control. It is unavoidable that this technology cannot turn off the magnetic field in the entire workspace or drive high‐frequency magnetic fields.^[^ ^77^ ^]^

When trying to integrate the stationary electromagnet control system and the permanent magnet control to create a mobile electromagnet control system, researchers discovered that the combination of electromagnetic coils can overcome the constraints of the aforementioned issues. The mobile electromagnet control system seeks to combine the benefits of the two methods to avoid their drawbacks.^[^ ^80^ ^]^ It has enough flexibility and the capacity to turn on and off the magnetic field. Additionally, the mobile electromagnet system may reduce the heat generated in the workspace and shorten the distance from the field source to the controlled microrobot when compared to the stationary magnetic system. The specific movement path of the movable magnetic system has yet to be designed, though. A mobile electromagnetic control system known as the DeltaMag system was created by Yang et al. and is capable of moving coils in parallel while commanding a microrobot to brake with 6° of freedom in 3D.^[^ ^81^, ^82^ ^]^ Three symmetrically dispersed coils of DeltaMag are capable of producing any magnetic field in 3D. Additionally, DeltaMag has a camera mounted on the plate that can follow the tiny robot in real time. Based on computer vision, three coils are regulated in a closed loop. Additionally, each coil must be accurately mathematically modeled, and the parallel mechanism affects how the microdevice moves. Planning the movement of DeltaMag is crucial because every time the microrobot moves, the magnetic field must be changed again. They also suggested a triple‐loop visual servoing system, which is employed for swimmer steering in addition to mechanism tracking. The ability of DeltaMag to do very accurate magnetic manipulation in expansive workspace with little current can help conserve energy. This is possible because it can travel toward the microrobot and follow it there. For vascular catheter testing and gastrointestinal (GI) endoscopy, this technology has immense potential.^[^ ^77^ ^]^

The potential biomedical applications of electromagnetic actuation systems for the manipulation of microrobots in blood vessels have also been studied,^[^ ^2^, ^83^, ^84^ ^]^ showcasing the navigational potential for diagnostic and therapeutic procedures. Overall, electromagnetic devices provide a versatile and effective means of actuating and controlling microrobots in diverse fields, ranging from medicine to microfluidics.

## 3 Electric Field Actuation

Micro/nanorobots can be controlled by external electric fields. Studies have demonstrated that the two main techniques for actuating robot movement are electro‐osmosis and electrophoresis.

Electro‐osmosis is the movement of liquid through a porous surface as a result of an applied potential, while electrophoresis is the flow of charged particles through a liquid or gel when an electric field is present. The strength and direction of these phenomena can be controlled to perform specific tasks such as transport and catalysis. To trap and detect target molecules, they could also be included in a solid‐state nanopore platform. This demonstrates the diverse range of applications for nanorobots. Nonetheless, it is impossible to disregard the challenge of precisely controlling direction and speed. Si et al. developed a theoretical model of a nanorobot with four single‐stranded DNAs (ssDNA) (deoxyribonucleic acid) mounted on a quadnanopore device for motion control.^[^ ^85^ ^]^ The four ssDNA of the robot can be individually engulfed by the nanopores when an electric field is present. As illustrated in Figure 7A–C, the nanorobot is made up of one carbon ball (the core of the nanorobot) and four ssDNA (the legs of the nanorobot). Four nanopores are drilled in the corners of a square in the center of the graphene membrane. When an external electric field was applied along the *z*‐axis, all four ssDNA strands were finally trapped one to one by the nanopores in the graphene membrane. The graphene membrane then serves as a substrate for the mobility of the nanorobot. The yellow atoms at the nanopore borders were chosen to have their charges changed so that the strength and direction of the electro‐osmotic flow could be well controlled. The well‐controlled nanorobot is anticipated to have a wide range of intriguing applications, including cargo transportation and nanomanipulation. Finite element analysis was utilized by Qu et al. to simulate the magnetic field produced by a coil assembly to more efficiently actuate nanorobots, as shown in Figure 7D–E.^[^ ^86^ ^]^ They also intended to research electromagnetic fields in the use of nanorobots. A 3D magnetic simulation was run concurrently to determine the strength of the magnetic force acting on a cylindrical nanorobot. The simulation results were validated, and its viability was established by experimental measurements. These findings provide benchmark values for the use of nanorobots equipped with electromagnetic actuators. Such benchmarks could potentially be applied to biomedical applications, including drug delivery and the manipulation of individual cells.

Introducing conductive materials into a nanorobot and then adjusting the surface charge of the robot or the electrochemical reaction on the interface through electric fields can also yield actuation. Zhang et al. proposed an interdigital microelectrode system shown in Figure 7F–H.^[^ ^87^ ^]^ When an AC electric field is applied, metal electrolyte spherical nanorobots constrained by hydrodynamics can be polarized along the center line of the electrode, and the movement speed can be controlled. In addition, the nanorobots moving in the same direction travelled along a single file, while the nanorobots moving in the opposite direction reoriented themselves and moved with each other. At high particle density, as the multibody interaction becomes more complex, turbulent aggregates are formed. The microspheres are of 3 μm in diameter which can be used in the future for applications such as drug delivery.

**Figure 6:**
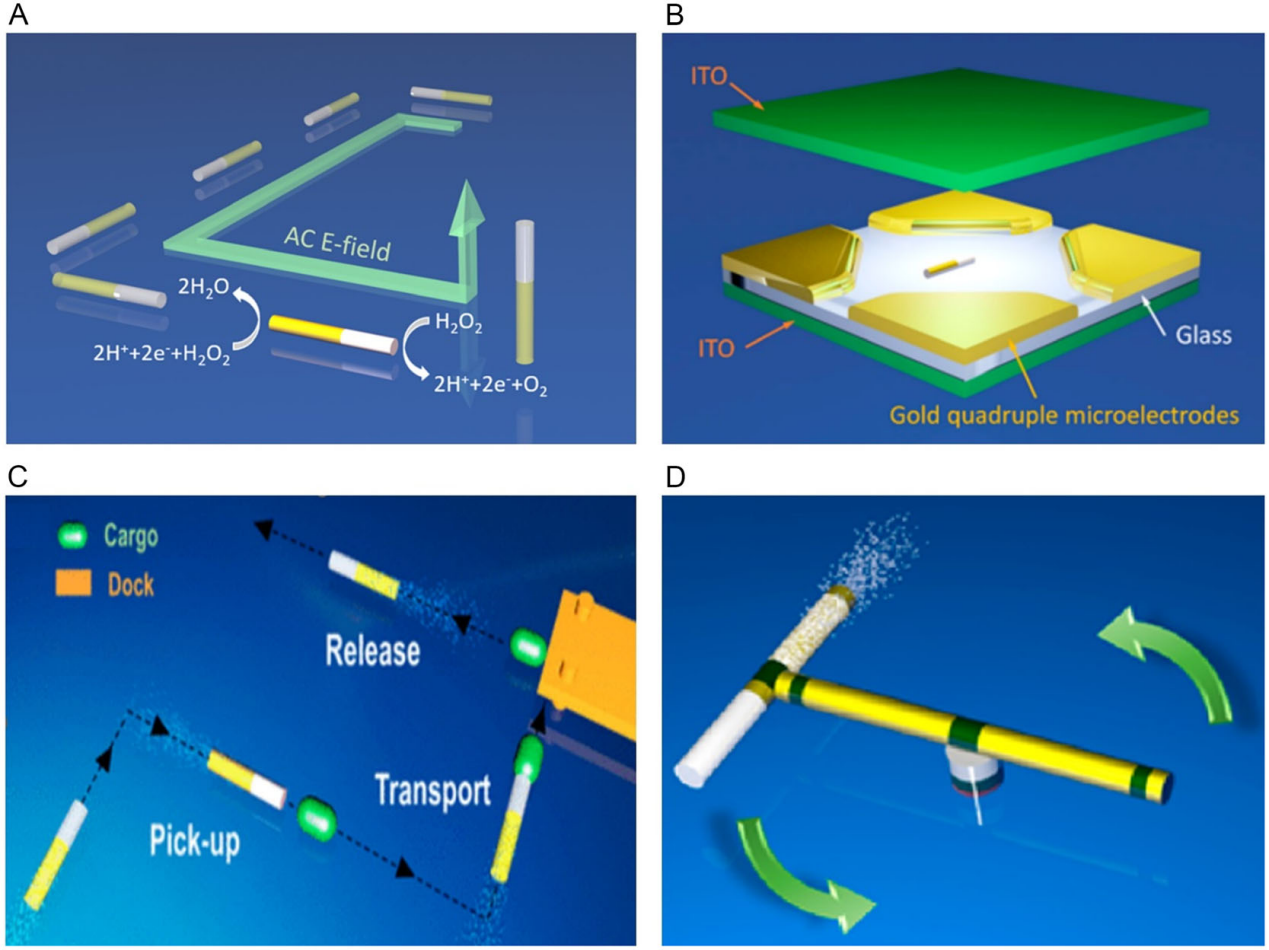
A) Schematic of 3D Pt/Au catalytic nanomotor manipulations in H_2_O_2_ fuel with AC E‐fields. B) 3D orthogonal microelectrode setup diagram (ITO being indium tin oxide parallel electrodes). C) Scheme of targeted cargo delivery. D) A schematic illustration of the rotating NEMS device, which is powered by a catalytic nanomotor. A–D) Reproduced with permission.^[^ ^88^ ^]^ Copyright 2018, American Chemical Society.

Using a 3D orthogonal microelectrode device to produce AC and DC electric fields, Guo et al.^[^ ^88^ ^]^ suggested a technique to operate nanomotors, which can precisely regulate the delivery of cargo such as in **Figure** **6**. They are 5 μm and 250 nm in length in diameter respectively. It travels at a speed of ≈12.5 μm s^−1^ with a rotation speed of 0.64 rad s^−1^. The AC electric field independently and precisely directs the nanomotor by electric torque on the dipole, while the DC electric field modifies the transmission speed through electrophoresis and electroosmosis effects. Both the loading and transportation of the cargo are made possible by the efficient combination of the two. This research findings encourage high‐precision velocity control and adjustment of nanomotors. This study may serve as an inspiration for the development of numerous nanorobots and useful nanoelectromechanical systems (NEMS)/micro‐electromechanical system (MEMS) instruments for a range of tasks in electronics and biomedical research.^[^ ^88^ ^]^

Robots and medical implants alike may maintain their shape without constant power thanks to shape‐memory actuators, which is a benefit in circumstances where the devices are untethered, and power is scarce. The requirement for micrometer‐scale electroshape‐memory actuators is still substantially unmet, especially those that can be powered by common electronics, despite recent work demonstrating shape‐memory actuators using polymers, alloys, and ceramics.

This was until Qingkun Liu and fellow Cornell researchers presented a novel class of micrometer‐scale, rapid, high‐curvature, low‐voltage (≈1 V), reconfigurable shape‐memory actuators.^[^ ^89^ ^]^ They work by electrochemically oxidizing and reducing a platinum surface, which induces strain in the oxidized layer and results in bending. They operate inside the electrochemical window of water, bending to the shortest radius of curvature of any electrically controlled micro actuator (≈500 nm), respond quickly (≈100 ms), and prevent bubble production associated with oxygen evolution. It has been shown that shape‐memory actuators can be utilized to build fundamental electrically reprogrammable microscale robot components. These include actuating surfaces, morphing metamaterials, origami‐based 3D structures, and mechanical memory components. In addition, these shape‐memory actuators could lead to creating microscopic robots, bioimplantable devices, and adaptive microscale structures.^[^ ^89^ ^]^

**Figure 7:**
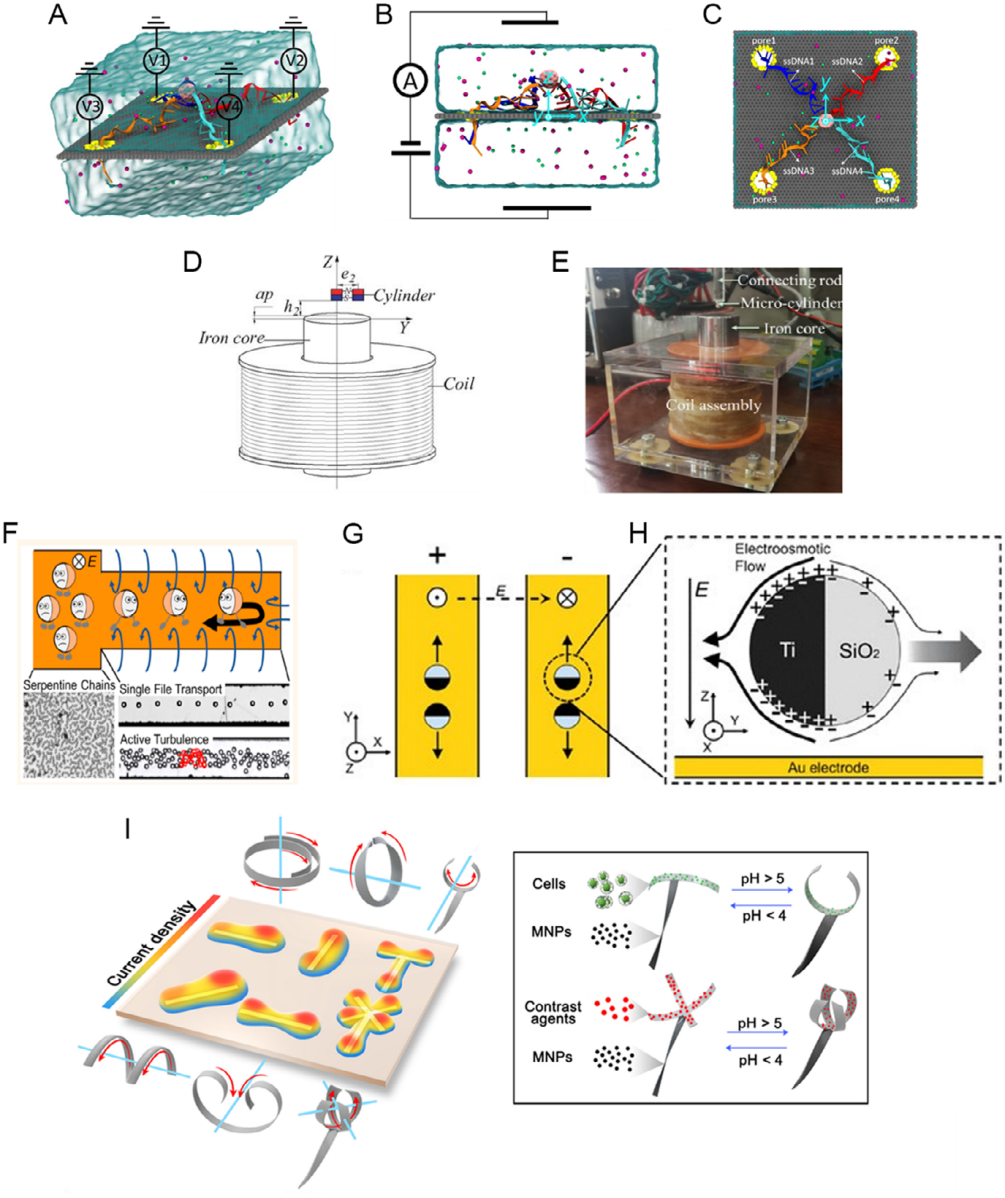
A) NP‐DNA assembled nanorobot. B,C) Schematic representations of its equilibrated simulation system from the side (B) and top (C). One C60 molecule (in pink) and four ssDNAs make up the nanorobot. The single‐layer graphene membrane atoms are depicted as gray spheres. The ionic solution is depicted as a transparent ice blue surface, with the potassium and chloride ions depicted as magenta and green spheres, respectively. The yellow atoms in the graphene membrane were chosen to be charged differently, resulting in varied surface charge densities for each nanopore. A–C) Reproduced with permission.^[^ ^85^ ^]^ Copyright 2020, American Chemical Society. D) Schematic of the coil assembly and the microcylinder. E) Image of the coil and microcylinder assembly. D,E) Reproduced with permission.^[^ ^86^ ^]^ The Electromagnetics Academy. F) An interdigitated microelectrode system in which the application of AC electric fields hydrodynamically confines and electrokinetically propels SiO_2_–Ti Janus particles in 1D along the electrode center lines with controllable speeds. G) A top view shows how the Janus particles traveling on electrodes are oriented and moving in that way. H) Schematic of an induced charge electrophoresis experiment on a SiO_2_–Ti particle. F–H) Reproduced with permission.^[^ ^87^ ^]^ Copyright 2019, American Chemical Society. I) Schematic representation Diverse types of shape morphing of MMRs generated through 3D electric fields. Drugs, contrast agents, cells, and MNPs can all be loaded by aniso‐electrodeposition into the various hydrogel microstructure modules of the MMR. Reprinted with permission.^[^ ^90^ ^]^ Copyright 2022, The Authors, published by AAAS. From ref. [90]. Copyright, The Authors, some rights reserved; exclusive licensee AAAS. Distributed under a CC BY‐NC 4.0 license http://creativecommons.org/licenses/by-nc/4.0/. Reprinted with permission from AAAS.

Moreover, Zheng et al.^[^ ^90^ ^]^ were able to develop a single‐step anisoelectrodeposition fabrication process for modular microrobots (MMRs) with individual functionalities, reaching a propulsion speed of 8.3 mm s^−1^. The microscale stripe‐shaped structure can be given a variety of shape‐morphing abilities (as shown in **Figure** **7**I), including spiraling, twisting, bending, and coiling, by manipulating the electric field. The suggested construction technique can create MMRs with numerous autonomous modules, which can then be loaded with cells, medications, and MNPs to achieve multifunctionality. MMRs may thus carry out numerous functions at once, including propulsion, gripping, and item delivery. Future applications for focused immune cell and drug administration as well as accurate tumor treatment may make use of the proposed delivery technology.

## 4 Light Field Actuation

The utilization of light as a means of actuation in the field of micro and nanorobotics has gained significant traction, highlighting remarkable potential for diverse applications, including theranostics^[^ ^91^ ^]^ and drug release mechanisms.^[^ ^92^ ^]^ Light actuation enables precise control over micro and nanorobots by harnessing the unique properties of light, such as its noncontact nature, high precision, and ability to manipulate objects at small scales. These capabilities have spurred innovative approaches in biomedical research and other domains.

Comparing light‐actuated micro and nanorobots with other sources of actuation, such as magnetic, ultrasound and chemical methods, reveals distinctive strengths and limitations. Magnetic actuation offers excellent control and manipulation capabilities, particularly in complex environments such as endovascular or human digestive tract for targeted drug delivery.^[^ ^93^ ^]^ Ultrasound actuation, on the other hand, allows for remote control as ultrasound waves can penetrate deep into tissues. For instance, Li et al. developed ultrasound‐responsive nanorobots that can be remotely guided and controlled for precise drug delivery within the GI tract.^[^ ^36^ ^]^ Chemical actuation provides the advantage of being triggered by specific biochemical cues such as oxygen, pH, and enzymatic changes within the body. Hu et al. demonstrated a chemical‐responsive microbots that release drugs in response to pH changes within the body, targeting areas with abnormal acidity, such as tumours.^[^ ^94^ ^]^ However, light offers a wide range of wavelengths, enabling tailored interactions with varied materials, making it a versatile and adaptable actuation mechanism.

**Figure 8:**
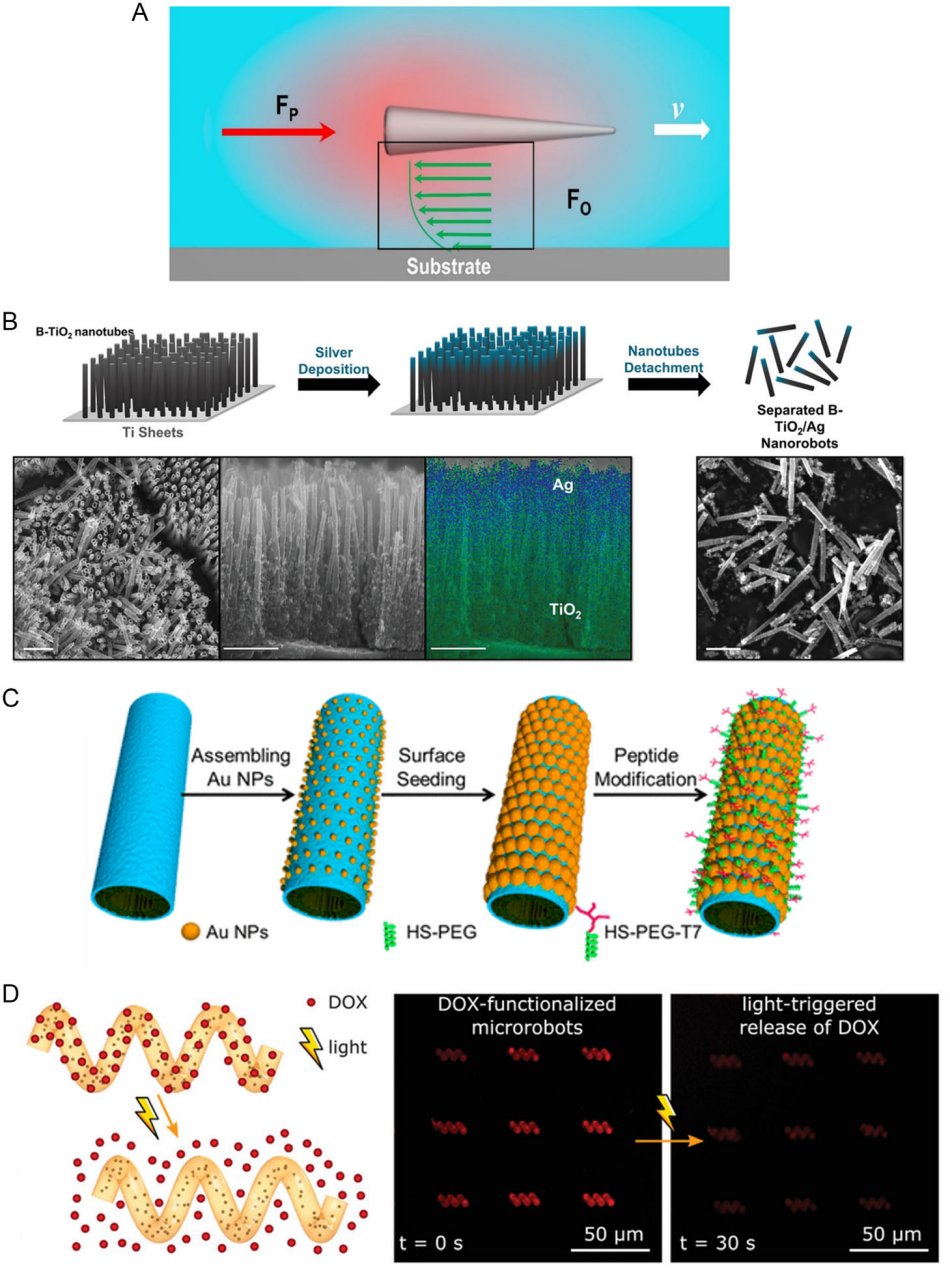
A) A controlled, needle‐like LMGNS that moves when exposed to NIR laser. Reproduced with permission.^[^ ^18^ ^]^ Copyright 2021, Elsevier. B) B‐TiO_2_/Ag nanorobot fabrication and characterization. The production processes of B‐TiO_2_/Ag nanorobots are depicted schematically, together with the matching cross‐view SEM–energy‐dispersive X‐ray spectrometer (EDX) picture and top‐view and cross‐view SEM images before and after the nanorobots detachment from Ti sheets. The scale bars are 2 μm long. Reproduced with permission.^[^ ^100^ ^]^ Copyright 2022, Wiley‐VCH. C) Schematic of The Pt NP‐modified polyelectrolyte multilayer manufacturing process of microengines. The polymer multilayer rockets use a NIR laser to irradiate microengines made of catalytic polymers to move on demand. Reproduced with permission.^[^ ^103^ ^]^ Copyright 2014, American Chemical Society. D) Schematic illustration of DOX‐functionalized carboxybetaine microrobots for UV‐light‐triggered drug release and an example for the drug release demonstration. Reproduced under the terms of the CC‐BY Creative Commons Attribution 4.0 International license (https://creativecommons.org/licenses/by/4.0).^[^ ^47^ ^]^ Copyright 2020, The Authors, published by Wiley‐VCH.

One notable application of light‐actuated micro‐ and nanorobots is in drug delivery systems. By incorporating light‐responsive materials into these robots, researchers have devised strategies for on‐demand drug release at specific locations within the body. For instance, in 2020, a study by Wang et al. conducted a study on the needle‐shaped liquid metal gallium nanoswimmer (LMGNS), which can be controlled under NIR laser irradiation.^[^ ^16^ ^]^ The developed light‐responsive nanorobot (shown in **Figure** **8**A,D) releases doxorubicin (DOX), a chemotherapeutic drug, upon exposure to near‐infrared (NIR) light.^[^ ^18^, ^47^ ^]^ The nanorobots were guided to tumor sites and triggered to release the drug, resulting in enhanced antitumor efficacy and reduced side effects.^[^ ^18^ ^]^ DOX delivery and release is used extensively in similar micro/nanorobots.^[^ ^95^, ^96^, ^97^, ^98^, ^99^ ^]^ This robot has prospects for use biomedical applications such as targeted delivery and active soft matter materials. Additionally, it was seen that the LMGNSs had a length of 7.07 ± 0.68 m and diameters of 823 ± 86 and ±53 nm, respectively, at their tail and tip ends. The light‐triggered shape changes in polymer‐based microrobots offer the ability to achieve controlled locomotion and manipulation at the microscale. The propulsion force of this nanoswimmer is generated by the thermophoresis force resulting from the temperature gradient along its longitudinal axis. The researchers found that the speed nanoswimmer is adjustable as a function of the light intensity, with a maximum speed of 31.22 μm s^−1^ achieved under a laser intensity of 5 W cm^−2^. In a similar study, Li et al. reported on a maximum speed twice that of previously developed nanoswimmers (59.6 μm s^−1^) using ultraviolet irradiation. However, the needle‐shaped LMGNS has the advantage of being more precise in targeting and safer to use as it employs NIR light, which is less likely to cause safety issues than UV light.^[^ ^100^ ^]^ An example of a UV light nanobot (shown in Figure 8B) was used for facial titanium implant removal. Titanium miniplates are biocompatible materials used to heal facial bone fractures in current oral and maxillofacial surgery. However, due to implant problems, plate removal is frequently required. Among these, the establishment of a biofilm on an infected miniplate is linked to significant inflammation, which frequently leads to implant failure. In light of this, innovative techniques for controlling or treating oral bacterial biofilm are highly sought after. Ussia et al.^[^ ^100^ ^]^ use nanorobots (maximum length of ≈3 μm) to imitate pathogenic conditions in the oral microenvironment by growing multispecies bacterial biofilm on commercial titanium miniplate implants. The technique relies on light‐driven self‐propelled tubular black‐TiO_2_/Ag nanorobots, which, unlike standard ones, have an extended absorption and motion actuation range from UV to visible light. By creatively using photoresponsive DNA (deoxyribonucleic acid) signaling molecules, Sato et al. created a molecular robot that resembled an amoeba.^[^ ^101^ ^]^ This DNA‐based nanorobot is entirely made of biological and chemical components, unlike conventional nanorobots. An actuator and a clutch are located inside a vesicle made of phospholipid bilayers that serve as the major structural component. The photoresponsive DNA will separate into single strands and cling to the microtubules when the robot is exposed to UV light. The outer cell membrane changes due to the sliding of microtubules, turning the robot from an inactive sphere to an active sphere moving nonsphere. In contrast, when the robot is exposed to visible light, the microtubes are unable to contact with the membrane, causing the clutch to disengage. As a result, the robot returns to its original spherical shape and comes to a stop. This study successfully modeled the motion of cells and offered a strong foundation for the creation of molecular robots. However, there are still limitations to consider, such as the unidirectional motion of DNA‐based robots and the inability to reverse the behavior‐changing process, which may be significant obstacles for future research.

Miskin et al. conducted innovative research on voltage‐controllable electrochemical actuators that operate at low voltages (200 μV), low power (10 nW), and are compatible with the current silicon processing.^[^ ^102^ ^]^ The creation of microscopic robots has long been constrained by the scarcity of microscale actuators. For millimeter‐sized robots, conventional piezoelectric actuators work well, but they are ineffective at the micrometer to nanoscale. As a result, the innovation consisted of creating a new form of electrochemical actuator and using it as the legs of robot. It was created using a common photolithography technique and was made of platinum NPs (Pt NPs). The actuator will flex and cause the robot to walk when it is illuminated by a sequence of laser pulses. The width and length of the robot bodies are 40 and 40 μm or 40 and 70 μm, depending on the number of photovoltaics mounted onboard, and the maximum thickness is 5 μm. Peak speeds were just under 30 μm s^−1^ and an average speed of about 1 μm s^−1^ or roughly 1 body length each minute.

Furthermore, NIR light is of special importance in the field of biomedicine due to its minimal absorption and ideal tissue penetration. The He group used NIR light‐responsive polymer as a microengine with a programmable “on–off” motion, shown in Figure 8C.^[^ ^103^ ^]^ The two openings have widths of around 5 and 7 μm, respectively, while the tube has length of roughly 10 μm. The microengines were created using a template‐assisted method and covered in a mixed layer of a tumor‐targeting peptide and antifouling poly (ethylene glycol). The microengines remain still at a critical concentration of peroxide, but NIR light activates their movement through a photothermal effect. The photothermal effect can also activate the engines without peroxide fuel, potentially eliminating the need for toxic fuel in the future. This was done through functionalization of gold nanoshell after layer‐by‐layer deposition of polymers and Pt NPs into the nanoporous template, which was assisted by a nanoporous template, to create the tubular nanorobots. The delayed production of oxygen bubbles prevented the swimming nanorobots from moving at the propulsion threshold concentration of hydrogen peroxide (H_2_O_2_) (0.1%). But when exposed to NIR light, the swimming microrobots became active and demonstrated effective chemical propulsion in the same concentration of H_2_O_2_ This NIR light‐induced on‐and‐off motion is ascribed to quicker mass transfer and improved catalytic reaction kinetics. The NIR laser‐controlled emission and self‐propulsion capabilities of these swimming microrobots can also be used for highly targeted cancer detection and subsequent photothermal cancer treatment.

Additionally, swimming microrobots can have their speed controlled by brief heat pulses. Pt/Au nanowires were observed to move much faster at high temperatures than at room temperature.^[^ ^104^ ^]^ The elevated velocity is related to the rise in electrochemical reaction temperature and the fall in solution viscosity. It is possible to obtain enhanced spatiotemporal navigation by combining electrochemical and magnetic guidance. Despite advantages of optical actuation, it also has limitations. One such limitation is its ability to penetrate nontransparent media deeply for a given wavelength of light. This poses a limitation for actuating microrobots in deep locations, such as inside the human body. However, larger‐wavelength light sources, such as NIR light, have a greater penetration depth under the skin, up to 1–2 cm. In contrast, magnetic actuation has the advantage of being able to penetrate any nonmagnetic media deeply. A clinical MRI scanner, for example, can cover a whole human body cross section with a 3 T uniform magnetic field and uniform magnetic field gradients in 3D, with no tissue penetration problems. The only limiting penetration depth constraint in this case could be the range of magnetic field generators, as magnetic fields decay exponentially with distance from the source location. Additionally, the limitations of light‐actuated microrobots and nanorobots in terms of biocompatibility and biodegradability can be summarized as follows.

*Biocompatibility*: The potential for adverse biological reactions or toxicity when interacting with living systems is biocompatibility.^[^ ^105^ ^]^

*Biodegradability*: The ability of light‐actuated microrobots to biodegrade within the body once their tasks are completed is biodegradability. Biodegradability is essential for minimizing long‐term foreign body responses and ensuring the elimination of microrobots from the body without the need for additional invasive procedures.

*Long‐Term Stability*: Light‐actuated microrobots should maintain their structural integrity and performance over time to ensure reliable and effective operation. Factors such as photodegradation, material fatigue, or changes in properties due to environmental conditions (e.g., temperature, humidity) may impact their long‐term stability, thereby limiting their practical use.^[^ ^106^ ^]^

*Scalability and Manufacturing*: The fabrication of light‐actuated microrobots with precise control over their size, shape, and properties can be challenging. Achieving scalable and reproducible manufacturing processes for these intricate systems is essential for their widespread use. Additionally, integrating light‐responsive components into micro/nanorobots without compromising their biocompatibility and biodegradability requires careful engineering and optimization.^[^ ^107^ ^]^ Addressing these limitations requires ongoing research and development efforts to enhance the biocompatibility, biodegradability, stability, and manufacturing techniques of light‐actuated microrobots. By overcoming these challenges, the potential for their applications in precision therapeutics, targeted drug delivery, and minimally invasive surgery can be further realized.

## 5 Acoustically Driven Micro/Nanorobots

Acoustically driven micro/nanorobots are a promising class of miniaturized robots that can navigate and manipulate at small scales, enabling a wide range of applications in medicine, biotechnology, and engineering.^[^ ^14^, ^108^, ^109^, ^110^ ^]^ The state of the art in this field involves developing new types of acoustic sources, materials, and designs that can enhance the precision, control, and functionality of these robots.^[^ ^111^, ^112^, ^113^, ^114^ ^]^

Acoustic waves have also been used for actuation, manipulation, and navigation of micro and nanorobots. Acoustic waves can be used to generate forces on the surface of the robot, which can be used to propel it or manipulate its position. One of the advantages of acoustic actuation is that it can be used in liquid environments, which makes it a promising method for applications in biology and medicine.

One notable approach is to use ultrasound waves to generate forces and torques on the micro/nanorobots. For example, researchers have demonstrated the use of ultrasound fields to manipulate microbubbles and microbeads, which can act as carriers for drugs or genes.^[^ ^115^ ^]^ Other studies have shown that acoustic tweezers can trap and move particles in a microfluidic channel, enabling cell sorting and tissue engineering applications.^[^ ^116^ ^]^ Another approach is to use piezoelectric materials to convert acoustic energy into mechanical motion, allowing for the design of autonomous micro/nanorobots that can swim or crawl in different environments.^[^ ^117^ ^]^ The field of acoustically driven micro/nanorobots is rapidly evolving, with new advances in design, fabrication, and control methods. With continued research and development, these tiny robots have the potential to transform the way we approach many different fields and challenges.

### 5.1 Focused Ultrasound

**Figure 9:**
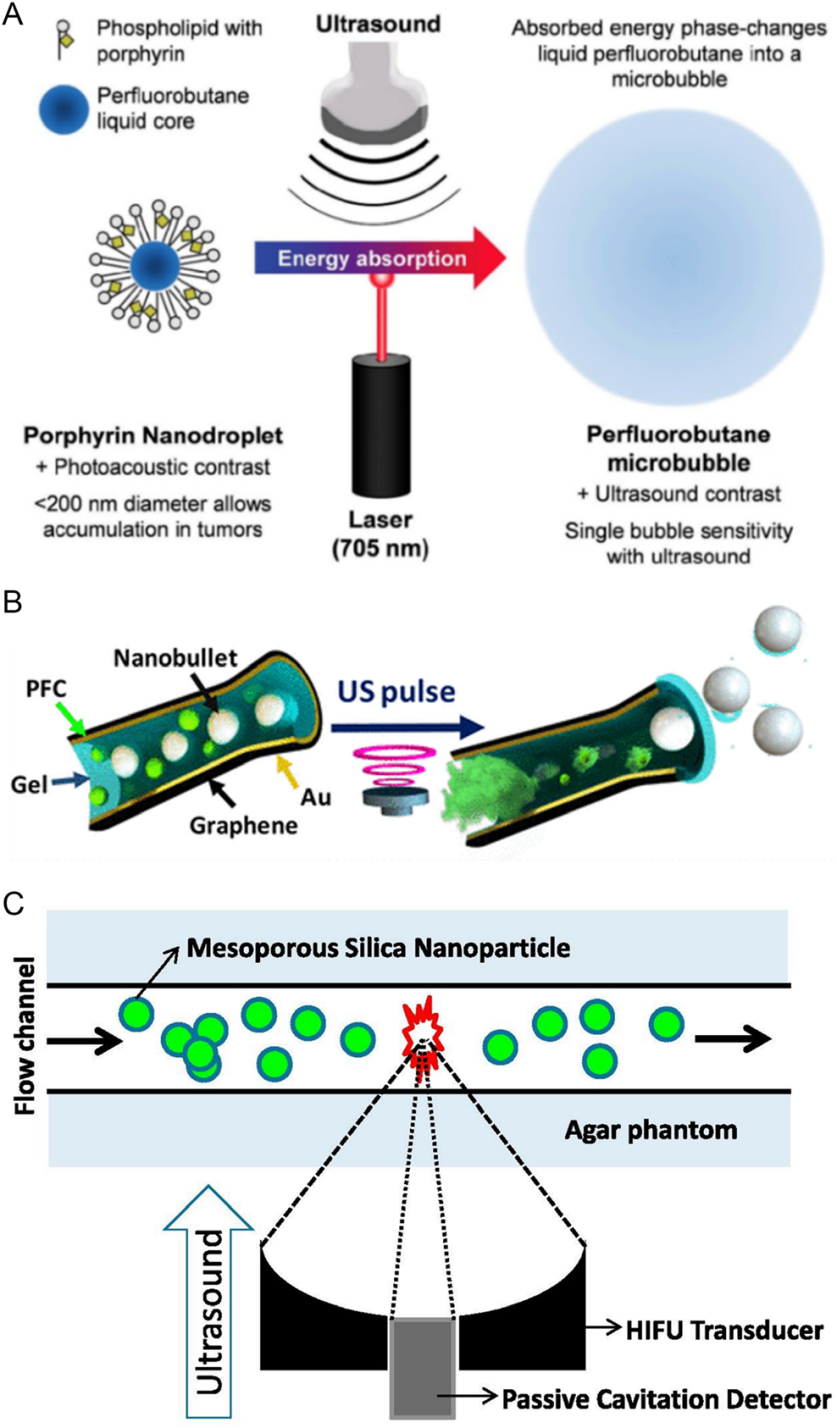
A) Phase‐change porphyrin nanodroplets into PFC microbubbles. Reproduced with permission.^[^ ^115^ ^]^ Copyright 2016, Wiley‐VCH. B) A microcannon firing NBs using PFC. Reproduced with permission.^[^ ^119^ ^]^ Copyright 2016, American Chemical Society. C) Scheme of fluorescein isothiocyanate‐labeled mesoporous silica NP delivery experiments in the agarose phantom model utilizing ultrasound‐mediated cavitation‐enhanced extravasation. Reproduced with permission.^[^ ^123^ ^]^ Copyright 2018, Elsevier.

Various types of micro/nanorobots have been powered and operated by focused ultrasound (FU). Researchers have employed transducers, such as concave, cylindrical, or arranged, that concentrate MHz waves with high pressures into a focal point to produce a focused ultrasonic field.^[^ ^118^, ^119^ ^]^ This pressure can be utilized to activate a bubble trapped inside a designed structure or to evaporate a liquid fuel. For instance, perfluorocarbon (PFC) emulsions can be made to evaporate rapidly when subjected to a high‐intensity FU beam. This process, known as acoustic droplet vaporization (as shown in **Figure** **9**A), causes a rapid transition from liquid to gas. The interior of the microstructure bubble rapidly expands, transferring mechanical energy to it and causing it to move in a bullet‐like manner at high speeds. Details of such bots are shown in **Table** **4**. The PFC emulsion is either embedded inside a hydrogel that fills the hollow tube (≈ 5 μm diameter), or it is functionalized electrostatically on its interior surface, according to the most widely used designs of this form of actuation. The latter design also makes it possible to fill the structure with useful payloads, such as nanobullets (NB), which will be shot at high speeds. The capacity of the bullet design to pierce, penetrate, and sever tissues has demonstrated a potential future for nanosurgery applications.^[^ ^120^ ^]^

**Table 4:** FU in micro/nanorobots

Structure	Size	Frequency	Pressure [MPa]	Speed [m s^−1^]	Application	References
Microbullet	8 and 40 μm	Pulse 44 us	1.6	6.3	Penetrate, deform, and cleave kidney tissue	[118]
Microcannon	1–5 μm	Pulse 10 ms at 2.25 MHz	1.9	Mc = 9	Tissue penetration of drugs	[119]
				nb = 42.2		
Nanocups	480 nm	0.5 MHz	2.5–3	–	Transport drugs to tumors	[191, 192]
NP	500 nm	0.5, 1.6 MHz	0.5–4	–	Tissue penetration and transport of drugs	[193]

Controlling the oscillation of a stable bubble trapped in an engineered nanostructure is a different method of producing motion utilizing focus ultrasonic waves, shown in Figure 9B. In this technique, the energy is used to cause the trapped bubble to expand and contract rather than for phase change. As a result, the pulsating bubbles create a microstream that appears as a jet or several vortices. Hollow sphere designs have been created to shield the bubble from external forces and constrain the microstreaming into a single direction to harvest such forces and generate motion.

Microstreaming and bubble explosion are two additional mechanisms that can be used to direct mechanical force into a target tissue using FU. For example, a hydrophilic porous polymer cup containing bubbles (500 nm in size) can be used to generate microstreaming and mechanical force. Nanorobots based on droplet evaporation are smaller in size and are better suited for applications requiring dispersion inside the body.

The nanocups, which are designed to travel through the body and only trigger at a specific spot for tissue penetration distribution, reflect this idea. However, there are some restrictions that must be overcome, such as the quantity of shots and the number of components that already exist in the experimental arrangement. Recent research has focused on enhancing the bubble cavitation modulation as well as the augmentation brought on by nanorobots concentration. Nevertheless, these techniques have some drawbacks, including a brief lifetime and erratic motion brought on by wave interference. When the location of interest is saturated with many structures, the size dependency of the structures results in a reduction of the interference effect.^[^ ^121^ ^]^

Acoustically powered micro/nanorobots have also shown promise for therapeutic delivery applications. For example, graphene oxide and gold microcannons filled with a gel matrix that stabilizes PFC and silica NB—the suggested drug delivery systems—were employed. As the structures can be extravasated and the medication delivered where it is needed, several studies also fill polymeric nanocups with pharmaceuticals and propose to use deep penetration to destroy tumors. While FU‐powered nanodevices have unique advantages such as tissue penetration and targeted drug delivery, they may not be suitable for continuous motion or surgery. In contrast, armored bubbles, or bullets, which have directed motion capabilities, are superior for surgical applications (shown in Figure 9C).^[^ ^122^, ^123^ ^]^

Based on the selective activation mechanism and the adaptability of the cargo loading capabilities, the FU robots are therefore used for precise applications. The many applications and intricate systems incorporated into novel designs will be advantageous. Future study should concentrate on creating techniques for the after‐use collection or the usage of biodegradable materials since these motors are typically composed of nonbiodegradable materials. Additionally, targeted binding antibodies could help in treating only diseased tissue, resulting in fewer side effects, by selectively functionalizing the micromotor outer surface.^[^ ^124^ ^]^

### 5.2 Standing Wave Ultrasound

**Figure 10:**
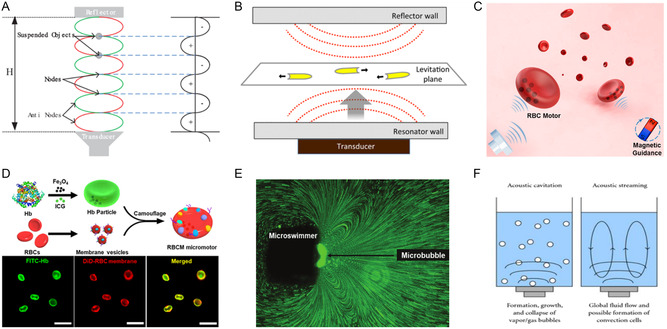
A) Acoustic radiation force schematic. Reproduced under the terms of the CC‐BY Creative Commons Attribution 4.0 International license (https://creativecommons.org/licenses/by/4.0).^[^ ^82^ ^]^ Copyright 2017, The Authors, published by Sage Publications. B) A schematic representation of the autonomous motion of axisymmetric gold nanorods in an acoustic cell. Reproduced with permission.^[^ ^117^ ^]^ Copyright 2016, American Chemical Society. C) Example of magnetically guided, ultrasound‐propelled RBC micromotors in whole blood. Reproduced with permission.^[^ ^125^ ^]^ Copyright 2014, American Chemical Society. D) Diagram of a camouflage fabrication technique for RBCM micromotors. Moreover, there are confocal laser scanning microscope images of Hb particle (green) camouflage by cell membranes (red). Scale bar is 5 μm. Reproduced with permission.^[^ ^132^ ^]^ Copyright 2019, American Chemical Society. E) Acoustic microstreaming flow generated by an asymmetrical oscillating bubble trapped within a microswimmer. Reproduced with permission.^[^ ^181^ ^]^ Copyright 2017, Wiley‐VCH. F) Schematic for acoustic cavitation and streaming visualization. Reproduced under the terms of the CC‐BY Creative Commons Attribution 4.0 International license (https://creativecommons.org/licenses/by/4.0).^[^ ^182^ ^]^ Copyright 2011, The Authors, published by Hindawi.

One of the most advanced methods for powering tiny microengines is through the use of standing wave ultrasound (SWU). This type of acoustic field is produced by the interference of ultrasonic waves traveling in opposite directions, resulting in the creation of a nodal plane. In a typical SWU arrangement, a piezoelectric transducer generates longitudinal waves that pass along the cavity (or reservoir) and are reflected by a glass slide, creating the interference pattern necessary for the standing wave. The height of the resonant chamber must be greater than half the wavelength of the applied mechanical wave to produce a standing wave. When ultrasound is used, it acts as the primary force that propels suspended microrobots into the nodal plane called as the levitation plane, defeating the effects of gravity. The asymmetric design of the microrobots causes local vibrational gradients and nonhomogeneous in‐phase oscillations inside the pressure node, allowing each structure to move independently. As a result, neither primary radiation (radiation coming directly from the source) forces nor dispersed waves are used as part of the propulsion system to propel the structure into a node. SWU can be visualized as shown in **Figure** **10**A.

According to theoretical models, when a vibrating motor particle is exposed to ultrasound, it causes microstreaming on the boundaries of the structure, which in turn produces propulsion. For microrobots with metallic components, Rayleigh radiation pressure (the mechanical pressure that is applied to any surface as a result of the exchange of momentum between a body and an electromagnetic field) is one of the key factors in autonomous propulsion. Additionally, it has been found that the structure of microrobot must have shape and/or density asymmetries to provide a preferential microstreaming direction that facilitates self‐propulsion.

Nanorods, cup shells, and most recently, Janus particles are popular designs for nanorobots with rods or wire‐like structures being the most researched for SWU propulsion. As shown in Figure 10B, these structures are typically created by template electrodeposition and have sharply asymmetric ends that produce the desirable microstreaming for propulsion.^[^ ^117^ ^]^

Recent studies have shown that augmenting the cavity using spheres in the template can increase the speed of nanorod by 50%, resulting in a new kind of nanomotor with effective propulsion and higher towing capacity, which demonstrated effective propulsion and a higher towing capacity than the nanorod construction due to the bigger practical diameter size of cavity. It was shown that increasing the density of coating material could improve propulsion. The ability of this concept to produce propulsion in many functional particles, such as liposomes as drug carriers, makes it extremely intriguing to researchers.

The capacity of nanorod robots to navigate through various biological media, including serum,^[^ ^125^, ^126^ ^]^ saliva,^[^ ^126^ ^]^ saltwater,^[^ ^126^ ^]^ blood,^[^ ^125^, ^126^, ^127^ ^]^ (shown in Figure 10C), and inside of cells, has also been exhibited. It was discovered that the viscosity of fluid affects their speed. Their speed can be adjusted by altering the voltage or power of ultrasound transducer, which modifies the microstreaming around the motors.^[^ ^102^ ^]^

Hybrid‐powered biological platforms can be strengthened with the acoustic propulsion characteristics and increased actuation mechanisms. A notable example of such are modified RBCs. Researchers organically formed RBC‐based multicargo‐loaded micromotor systems.^[^ ^128^ ^]^ The RBC micromotors were all coencapsulated into MNPs, quantum dots (QDs), and the anticancer medication DOX. The direct observation of their loading into the RBC motors at two different wavelengths is made possible by the fluorescence of both QDs and DOX. The possibilities of MRI and effective magnetic navigation under ultrasonic propulsion are both made by the existence of MNPs in RBCs. The RBC micromotor performance is barely affected by the simultaneous encapsulation of therapeutic payloads and imaging NPs. Additionally, the RBC micromotors reveal the capacity to transfer imaging and therapeutic chemicals swiftly and precisely over a sophisticated microchannel network with less toxicity detected after drug encapsulation within the motor. Other RBC‐based micro/nanorobots have since been developed.^[^ ^129^, ^130^, ^131^ ^]^ One of the most notable ones is acoustically powered and magnetically navigated RBC‐mimicking RBCM micromotor developed by Goa et al.^[^ ^132^ ^]^ capable of actively delivering oxygen and photosensitizers (PS) for improved photodynamic cancer therapy.

RBCM micromotors (shown in Figure 10D) are made up of biconcave RBC‐shaped magnetic haemoglobin cores that encase PSs and natural RBC membrane shells. When exposed to an acoustic field, they can move in biological media at up to 56.5 μms^−1^ (28.2 body lengths per second). An external magnetic field can be used to navigate the direction of these RBCM micromotors. One method that has shown to be effective for RBC micro/nanorobots specifically is RBC hitchhiking.^[^ ^133^, ^134^, ^135^ ^]^ Medication administration using RBCs can significantly raise the medication concentration in the target tissues. When drug‐loaded NPs are delivered intravenously after adhering to RBC, also known as hitchhiking, the NPs go to cells of the capillaries in the downstream organ. It has been shown that RBCs can travel within various animals and organs.

Standing wave nanorobotics shows great promise in biomedical applications, as their fabrication methods are straightforward, and they can be functionalized with various receptors for a wide range of functions such as targeted drug administration,^[^ ^136^ ^]^ detoxification,^[^ ^137^ ^]^ and internalization in cells.^[^ ^138^ ^]^ While achieving SWU inside the body is challenging, this method is ideal for in vitro and lab‐on‐chip applications, enabling the testing of novel medications, treatments, and nanosurgeries in carefully controlled artificial biomedical settings.

### 5.3 Traveling Wave Ultrasound

To understand traveling wave ultrasound (TWU), we must understand the concept of acoustic microstreaming (Figure 10E). This occurs when an oscillating flow creates a nonlinear, second‐order steady flow, and viscous effects are concentrated along walls in a boundary layer. The streaming usually occurs in a rapid vortex, and it can be observed using various techniques such as UV photopolymerization, multistep electrodeposition, lithography, and 2PP. Rayleigh first proposed the idea of acoustic microstreaming in 1884, but Faraday was the first to observe it. Rayleigh showed that an oscillatory flow of velocity *U*(*x*)cos*t* along the wall produces a steady‐state streaming flow of velocity *u*_s_ along the wall (*x* being a coordinate along the wall). To be precise, this only occurs if the streaming Reynolds number *u*_s_ *ρ*_L_ *l*/ *u*_L_ is significantly less than 1 (*l* being the typical scale over which streaming occurs). Streaming usually occurs in a rapid vortex as shown in the Figure 10F.^[^ ^139^ ^]^

When compared to the template approach used to create the wire, cup, and hollow tube designs used by standing wave and focus ultrasound nanomotors, the employment of such a variety of techniques enables us to create complicated geometries.^[^ ^114^ ^]^

The usage of TWU is an innovative strategy for the creation of the next generation of nanorobots since it eliminates the need for a wave arrangement in the system to produce propulsion. This is particularly important for in vivo biological applications. The major benefits of this kind of nanorobots are their excellent tunability at specific operational frequencies and its adaptability to various propulsion configurations. The precise motion control of motors over arbitrary surfaces, 3D navigation, and capacity for nano‐object manipulation and microstreaming mixing are among its key features. Furthermore, the production of these structures using lithography techniques allows for high levels of reproducibility and scalability. Additionally, new advanced and active materials, such as luminescent materials, piezoelectrics, and biomaterials, will be simple to incorporate. Numerous applications, including nanosurgery, cell manipulation, cargo trapping, medication administration, sensing, transport, and others, can result from this. However, their development and delivery to the desired size remain a challenge in current research, with only a few examples reaching that level. Nonetheless, they are an exciting and evolving field with tremendous potential for the future.^[^ ^118^ ^]^

## 6 Chemical Actuation

**Figure 11:**
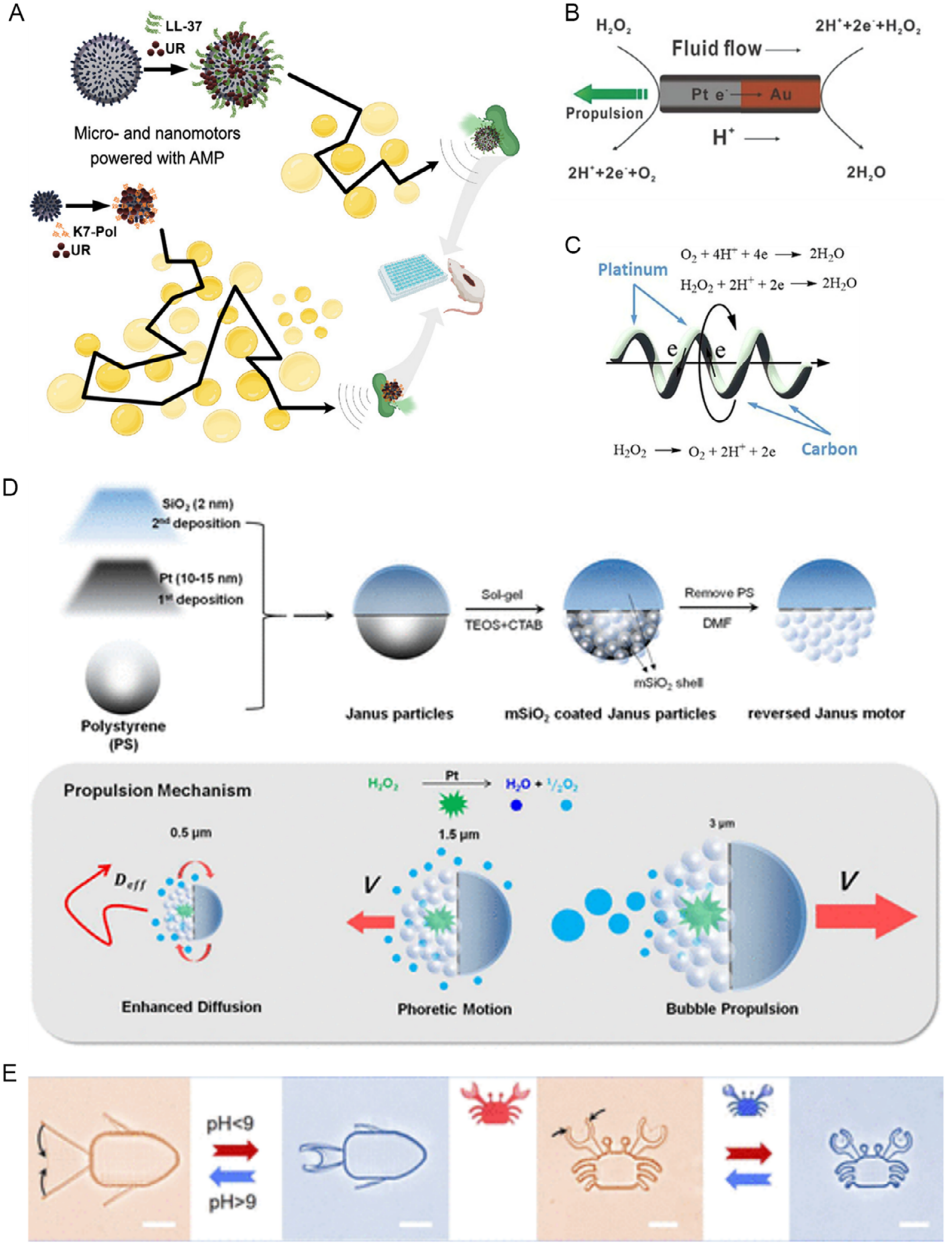
A) Schematic illustration of bioactive micro‐ and nanomotors coated with antimicrobial peptides (AMP) for the autonomous infection treatment. Based on the AMP‐coating process of the urease micro‐ and nanomotors, the micro‐ and nanomotors can be propelled autonomously to target pathogenic infections both in vitro and in vivo. Reproduced with permission.^[^ ^142^ ^]^ Copyright 2022, American Chemical Society. B) Au–Pt bimetallic nanorod propelled by a self‐electrophoresis mechanism in an aqueous H_2_O_2_ solution. Reproduced with permission.^[^ ^144^ ^]^ Copyright 2018, Wiley‐VCH. C) Diagram displaying the propulsion of catalytic helical carbon motors system. Reproduced with permission.^[^ ^145^ ^]^ Copyright 2019, Wiley‐VCH. D) Diagrammatic illustration of the process steps involved in producing reversed Janus motors and the various mobility scenarios. Reproduced with permission.^[^ ^146^ ^]^ Copyright 2016, American Chemical Society. E) SMMC and SMMF optical pictures showing, respectively, how pH variations cause the fins and claws to open and close. Reproduced with permission.^[^ ^95^ ^]^ Copyright 2021, American Chemical Society.

Chemically or biochemically driven nanorobots typically consist of an active metal catalyst and an inert substance.^[^ ^140^ ^]^ The inert material is employed to create an asymmetric structure, while the catalyst/active metal is used to start a chemical/biochemical reaction at their surface (as illustrated in **Figure** **11**A).^[^ ^141^, ^142^ ^]^ Building an asymmetric field across NPs is crucial for designing chemically/biochemically propelled nanorobots. Janus particles, multilayer tubes, and bimetallic nanorods are examples of chemically and biologically driven nanorobots that have been developed. These nanorobots can create bubbles (matching the so‐called bubble recoil mechanism) or create a concentration gradient via self‐phoretic (phoretic indicating migration or movement of particles or electrons on the body) mechanisms, depending on the reaction involved. These nanorobots can be propelled by a variety of fuels, such as H_2_O_2_, urea/glucose, water, or acids and bases. H_2_O_2_ is reported to be the most frequently utilized fuel for synthetic autonomous nanorobots. There are two types of H_2_O_2_‐consuming autonomous nanorobots, each with a bubble‐driven or self‐phoretic mechanism. Paxton et al. created Au–Pt bimetallic nanorods (shown in Figure 11B) by electrochemically depositing the appropriate metals on alumina membranes, and they saw that the nanorods moved on their own in a solution.^[^ ^143^ ^]^ They are 370 nm in diameter, with Pt and Au segments that are 1 μm long each and can go up to speeds ten times their body length. Later, they verified self‐electrophoresis involving electrochemical H_2_O_2_ breakdown at both the Au and Pt ends. This mechanism describes the flow of electrons and protons within the bimetallic nanorod as they function as a short‐circuited galvanic cell. In this cell, H_2_O_2_ is reduced at the Au end, which acts as the anodic section, while H_2_O_2_ is oxidized to form O_2_ preferentially on the Pt end, which serves as the cathodic section. However, the self‐electrophoresis propulsion system of such nanorod robots produces a weak driving force. Moreover, high concentrations of H_2_O_2_ have intense oxidation, which is thought to be harmful to living things. To realize practical applications, particularly in vivo applications of chemically powered nanorobots in a biological system, it is vital to identify new in situ fuels other than H_2_O_2_. Following this cue, it is agreed that using biological fluids as a fuel source for nanorobots is preferable to using external fuels to keep the biological target alive.^[^ ^144^ ^]^ A nanorobot based on platinum (Pt) catalysis (shown in Figure 11C) was developed by Xu et al. and can be controlled by an electric mechanism that breaks down H_2_O_2_.^[^ ^145^ ^]^ This discovery holds promising applications in the biomedical or environmental field. They have also investigated the motion behavior associated with the number of rotations. The nanorobot could be able to overcome more resistance as it moves with an increasing number of rotations. This study also demonstrated that the movement can be influenced by the H_2_O_2_ concentration and Pt distribution. Despite the high yield provided by chemical vapor deposition, a fresh angle on future study will focus on how the oxide created during the process decreases the effective controllability. Future efforts should concentrate on amassing several controlled spiral motors.

Researchers reported on an inverted Janus structure of an internal catalytic “chemical engine” for applications such active cargo delivery (as shown in Figure 11D **)**.^[^ ^146^ ^]^ The mesoporous silica (mSiO_2_)‐based hollow particles include an embedded catalytic material, in this case Pt, which, when suspended in an aqueous peroxide (H_2_O_2_) solution, causes the H_2_O_2_ to decompose. Chemical species in solution can move between the outside and inside of the particle, thanks to the pores/gaps at the noncatalytic Pt hemisphere. They found size‐dependent motile behavior as improved diffusion for 500 nm particles and self‐phoretic motion, toward the nonmetallic region, for 1.5 and 3 μm particles as a result of changing the particle diameter. A theoretical model based on self‐phoresis explained the direction of motion. For the 3 μm particles, it has been found that the morphology of the porous section changes along with the mechanism of propulsion via bubble nucleation and ejection and the direction of motion.^[^ ^136^ ^]^

Another innovative technique behind chemical actuation is pH responsive. To treat localized cancer cells, Xin et al. programmatically encoded various expansion rates in a pH‐responsive hydrogel, creating shape‐morphing microrobots (SMMRs) that can change shape in response to their environment (as shown in Figure 11E).^[^ ^95^ ^]^ A shape‐morphing microcrab (SMMC) is able to deliver specific microparticles by grasping, carrying, and releasing them by “opening–closing” their claws in tandem with magnetic propulsion. A shape‐morphing microfish (SMMF) is created to encapsulate DOX by closing its mouth in phosphate‐buffered saline, pH 7.4, and release the medication by opening its mouth in a slightly acidic solution (pH 7). This is done as a proof‐of‐concept demonstration. The average speed of the SMMRs (10–100 μm) reached 60 μms^−1^ with morphing showing a fast response speed (≈600 ms).

## 7 Biological Actuation

**Figure 12:**
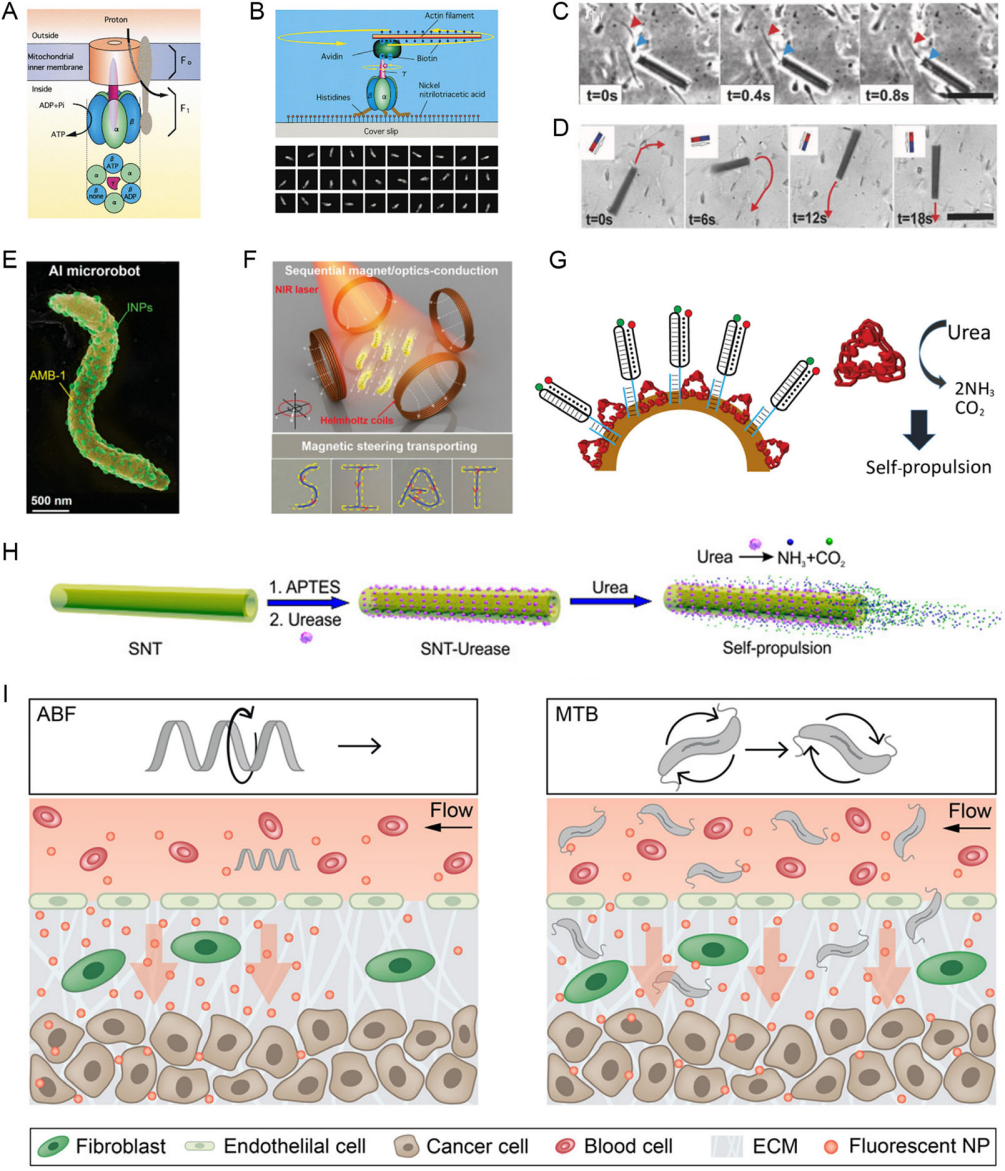
A) Simplified schematic of ATPase motor. F1 consists of *α*3*β*3*γδε* (*δ* and *ε* are not shown). The gray portion indicates a suggested location of *δab2*.) B) Visualization of F_1_ rotation. A,B) Reproduced with permission.^[^ ^183^ ^]^ Copyright 1998, Elsevier. C) The coupling of a bull spermatozoon inside a 50 μm‐long microtube. The sperm cell swims to the microtube (*t* = 0 s) and enters one opening of the tube (*t* = 0.4 s). The microtube made of thinly rolled titanium and iron nanomembranes. Red arrows point at flagella, whereas blue arrows point at the sperm head. Scale bar: 50 μm. D) Control of a sperm cell‐containing magnetic microtube by an external permanent magnet. Red arrows indicate a forward motion. Scale bar: 50 μm. C,D) Reproduced with permission.^[^ ^151^ ^]^ Copyright 2013, Wiley‐VCH. E) Schematic representation of successive magneto/optics‐conducted biohybrid microrobots (AMB‐1 microrobot paired with INPs, referred to as AI microrobot). It is an illustration of an AI microrobot as shown in an SEM image. (pseudo‐colored green, INPs; pseudo‐colored yellow, AMB‐1). F) Illustration depicting the AI microrobots in sequential conduction beneath magnetic/optical fields; bottom: complex 2D magnetic steering AI microrobot transporting routes. The motion images were captured using an optical microscope and a 40× objective lens in a Helmholtz coil setup. E,F) Reproduced with permission.^[^ ^154^ ^]^ Copyright 2020, Wiley‐VCH. G) The complementary DNA scaffold which is covalently linked on the micromotor can be hybridized by the pH‐responsive DNA nanoswitch. For self‐propulsion, urease enzymes described as red triangles are used to mediate the conversion of urea into ammonia and carbon dioxide. Reproduced with permission.^[^ ^158^ ^]^ Copyright 2019, American Chemical Society. H) Schematic of the construction of urease‐conjugated silica tubular (SNT‐urease) nanojets. Reproduced with permission.^[^ ^162^ ^]^ Copyright 2016, American Chemical Society. I) Schematic illustration of magnetically controlled micropropellers for convection‐enhanced NP transport. (left) A single microrobot, the artificial bacterial flagellum, designed for improving mass transport of NPs at the vessel–tissue interface. (right) Swarms of MTB to generate convective flow to enhance mass transport. Extracellular matrix: Reproduced with permission.^[^ ^165^ ^]^ Copyright 2019, The Authors, published by AAAS. From ref. [65]. Copyright, The Authors, some rights reserved; exclusive licensee AAAS. Distributed under a CC BY‐NC 4.0 license http://creativecommons.org/licenses/by-nc/4.0/. Reprinted with permission from AAAS.

Biological actuation is a field that explores how natural biological systems can be harnessed to create mechanical motion. A notable example is the F_0_F_1_ ATP synthase, also known as the ATPase motor (shown in **Figure** **12**A,B), which is a common rotational motor present in all living things. The mechanism of oxidative phosphorylation, one of the critical processes by which human bodies consume, handle, and extract energy from food, was addressed by Boyer.^[^ ^147^ ^]^ The ATP is a key player in this process. As demonstrated for the first time in the study by Carlo et al. the subunit rotation in the ATPase motor was in accordance to be used with the motors dimensions as well as the force formation in the molecular motors, which could be useful in the construction of nanoscale mechanical‐inorganic devices.^[^ ^148^ ^]^ A 1 nm diameter subunit rotated inside a 5 nm diameter F1 subunit, over 40 pN nm of rotating torque was observed, demonstrating the structure of the ATPase motor and its performance. Through experimentation and observation of the doping process of metals onto amino acids in the protein motor, Frasch et al. conducted a study in which they examined how the energy based on the ATP hydrolysis process is transformed into the physical force to pump a proton in a particular direction.^[^ ^149^, ^150^ ^]^

Magdanz et al. proposed the use of spermatozoa with flagella as an actuation force for micromanipulation and targeted drug delivery (shown in Figure 12C,D) for creating micro/nanorobots.^[^ ^151^, ^152^ ^]^ When combined the sperm cells and nanotubes, the swing motion of the sperm flagella interacts with the microtubes, causing the robot to move. Sperm‐driven micro/nanorobots are anticipated to be an effective tool in the field of artificial insemination and other reproductive technologies due to the high motility and safety advantages of spermatozoa over bacteria and other micro‐organisms.

Furthermore, Martel et al. discovered that there was no information regarding MC‐1 magnetotactic bacteria (MTB) in the treatment of cancer in earlier studies, particularly as an additional way to advance in smaller capillaries.^[^ ^153^ ^]^ To force therapeutic chemicals and microbeads together for targeted therapy, Martel creatively employed MC‐1 MTB along with ferromagnetic materials. According to the studies, using MTB to cure cancer is an efficient strategy, and further research exploring this technique may provide significant therapeutic potential. AMB‐1, a marine‐derived MTB, shown in Figure 12E,F, was employed by Xing et al. to develop the AI microrobot.^[^ ^154^ ^]^ Inspired by mice, magnetically controlled navigation was made possible using magnetic/optical sequential manipulation. A single robot or a group of robots can be moved precisely down to the micrometer scale while being tracked in real time throughout the body using dual‐mode fluorescence and MRI. The micro/nanorobot carried a PS and used magnetic targeting to penetrate complicated physiological barriers to infiltrate tumors. After that, the tumor was successfully treated using a NIR laser to produce a localized area of high temperature. This technology, which is now at the top of its field, will pave the way for future studies on micro/nanorobots with autonomous actuation.

Due to their fast reactions and availability of a variety of enzyme/fuel combinations, enzyme‐based *m*‐bots offer a fresh approach to the promotion of biocompatible self‐propelled *m*‐bots for biomedical applications Enzyme‐based *m*‐bots, one of the unique self‐propelled *m*‐bots, exhibit excellent activity and biocompatibility using the enzymes as catalysts instead of metals like Au, Pt, Ag, and MnO_2_. A self‐propelled Janus nanorobot was created by Sanchez and his team^[^ ^155^ ^]^ using hollow, mSiO_2_ NPs that were then used to drive biocatalytic reactions based on the three distinct types of enzymes that were fixed to the surface of the Janus NPs: catalase, urease, and glucose oxidase (as illustrated in Figure 12G.^[^ ^156^, ^157^, ^158^, ^159^ ^]^ The active nanomotors built on biocompatible enzymes introduced the possibility of utilizing biologically benign fuels for biomedical purposes.^[^ ^157^ ^]^ Several studies have confirmed that after being fastened onto synthetic micromachines like carbon nanotubes and tubular microrobots, enzymes demonstrated propulsion capability.^[^ ^160^, ^161^ ^]^ Bubble‐free tubular nanojets (220 nm diameter on average) using urea as the mild fuel were proposed by Ma et al. shown in Figure 12H, and propelled by an enzyme‐triggered biocatalytic reaction.^[^ ^162^ ^]^ Tubular nanojets (220 nm diameter) are promising for use in the biosystems because of their superior biocompatibility and longitudinal self‐actuation. Most recently, nitric‐oxide‐driven nanomotors constructed of hyperbranched polymide/l‐arginine were created by Wan and co‐workers.^[^ ^163^ ^]^ The amino acid l‐arginine was converted to produce NO. The difficulty in this area is combining controlled propulsion, biological *m*‐bots, and numerous benign fuels into an uncomplicated design that will allow the actuation and control of the *m*‐bots in various biological media without the *m*‐bots changing.^[^ ^164^ ^]^ Finally, some of the most important developments in the field of nano/microbot biological actuation are when used alongside magnetic in a hybrid actuation system.^[^ ^164^, ^165^, ^166^, ^167^ ^]^ an example described in Figure 12I.^[^ ^165^, ^166^, ^167^ ^]^

## 8 Hybrid Actuation Mechanism

Hybrid actuation mechanisms are a promising area of research for the development of micro‐ and nanorobots.^[^ ^168^, ^169^ ^]^ The use of magnetic fields and acoustic waves for actuation, manipulation, and navigation of micro‐ and nanorobots has been an active area of research in recent years.^[^ ^170^, ^171^ ^]^ While the field is still evolving, there have been several advancements in this area. Magnetic actuation is the most commonly used method, which then creates the foundation for developing hybrid actuation using biological, chemical, or physical methods. Recently, there has been noteworthy progress in the development of hybrid techniques that combine magnetic and acoustic actuation to achieve more precise control over the movement of micro and nanorobots. These hybrid techniques can take advantage of the strengths of both magnetic and acoustic actuation, while minimizing their limitations.

**Figure 13:**
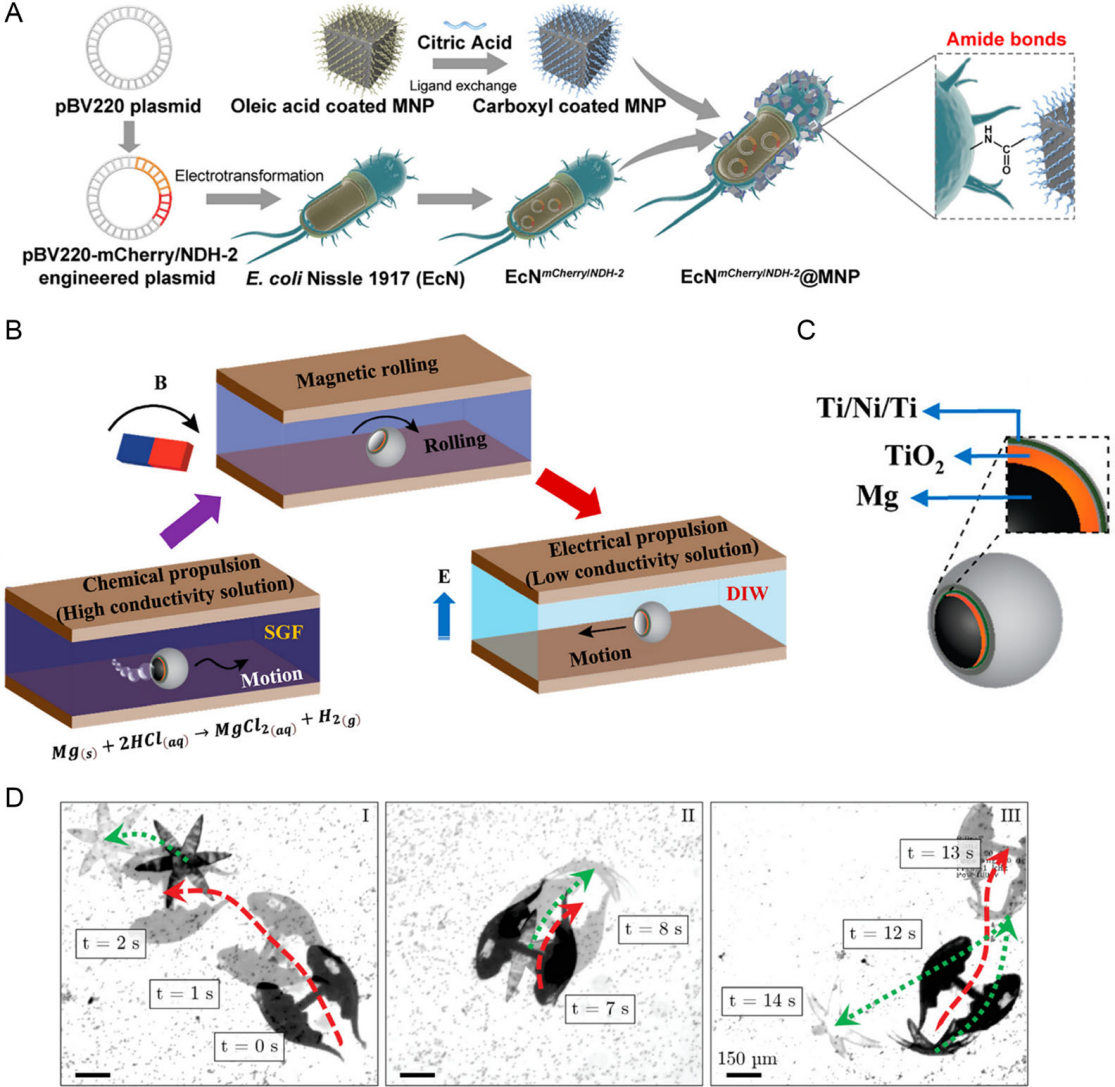
A) Schematic showing bacteria‐hybrid microrobots being prepared. Reproduced with permission.^[^ ^172^ ^]^ Copyright 2022, American Chemical Society. B) Hybrid propulsion mechanisms that use chemical, magnetic, and electrical bases to propel Mg‐core consumed particles in various solution conductivities. For chemical/magnetic propulsion, the experimental chamber consists of two parallel raw glass slides; for electrical propulsion, it consists of two parallel indium tin oxide (ITO)‐coated glass slides. C) The Mg‐core active particle (micromotor) structure that was used in the experiment. B,C) Reproduced with permission.^[^ ^173^ ^]^ Copyright 2022, American Chemical Society. D) Microrobot (indicated with red arrows) grabbing and releasing a star‐shaped microagent as payload, with time stamps showing the microrobot approaching the agent with its channel opening (I), pushing the agent toward the channel exit (II), and releasing the agent with a power‐modulated flow (III). Finally, after being released (*t* > 13 s), the microrobot manages to go away. D) Reproduced under the terms of the CC‐BY Creative Commons Attribution 4.0 International license (https://creativecommons.org/licenses/by/4.0).^[^ ^174^ ^]^ Copyright 2022, The Authors, published by Wiley‐VCH.

The biohybrid microrobot presented by Chen et al.^[^ ^172^ ^]^ highlights a remarkable integration of multiple functionalities crucial for effective targeted cancer treatment (**Figure** **13**A). With a speed of 0.42 mms^−1^ by harnessing magnetic, thermal, and hypoxia sensitivities, along with an internal fluorescent protein for dual reporting, this microrobot demonstrates an ability to navigate and respond to its environment in a collective manner for targeted cancer treatment. Central to this microrobotic system is the utilization of MNPs within probiotic *Escherichia coli* Nissle1917 (EcN@MNP). This innovative integration of EcN serves a dual purpose, enabling spatial magnetic manipulation and hypoxia perception. Moreover, a thermal‐logic circuit ingeniously engineered into the EcN bacterium regulates the biosynthesis of mCherry, which functions as a temperature and positioning reporter, adding a layer of precise control and feedback to the operations of the microbot. Additionally, the inclusion of the NDH‐2 enzyme encoded within EcN contributes significantly to enhanced anticancer therapy outcomes. The performance of the microrobot is further validated by its ability to actively target tumor regions in a collective manner under the influence of a magnetic field, as evidenced by fluorescent protein‐based imaging feedback. This dynamic functionality ultimately leads to the efficient induction of cancer cell apoptosis, both in vitro and in vivo, through the combined effects of magnetothermal ablation and NDH‐2‐induced reactive oxygen species damage.

A research team investigated electric fields combined with previously studied magnetic fields to develop a triple‐engine hybrid (electrical, chemical, and magnetic) micromotor for operating these micromotors (Figure 13B,C).^[^ ^173^ ^]^ This was done to address and prolong the finite lifetime of Mg‐based micromotors due to the depletion of the engine (Mg core) for targeted delivery applications. The core of the micromotor accounted for almost all of it size (other layers 100 nm) at 20 ± 5 μm. Electric fields are emerging as a promising energy source with many benefits. They are not time constrained and can easily adjust the frequency and amplitude of the field to dynamically regulate the mobility of the micromotor. Additionally, drug delivery as well as cell movement and trapping might both be accomplished using the same electrical fields.

However, these propulsion methods had limitations, especially in the low‐pH (and high‐conductivity) environment needed for Mg dissolution. Under these circumstances, electrical propulsion becomes quenched since it needs low‐conductivity media. Magnetic rolling was used in conjunction with magnetic steering as a way of self‐propulsion to move through the transition between these extreme and medium circumstances. It is interesting that electrical propulsion also required at least some Mg to be consumed, which gave the micromotor enough geometric asymmetry. The research team successfully used magnetic rolling inside a microfluidic device with the concentration gradient of the simulated stomach fluid to demonstrate the quick propulsion switching capacity of the micrometer, converting from chemical to electrical motions. For in vitro investigations simulating stomach circumstances and carrying out various bioassay activities, such triple‐engine micromotor propulsion offers a lot of promise.

CeFlowBot is a 420 μm × 500 μm magnetoacoustically driven microrobot developed by researchers for targeted drug deliveries with inspiration from aquatic organisms (Figure 13D).^[^ ^174^ ^]^ The Cephalopoda family, including octopuses, squids, and cuttlefish, exhibit a remarkable capability to draw in the surrounding fluid and expel it in a single‐jet thrust for propulsion. This intriguing biological phenomenon has inspired the development of acoustically powered microsystems that mimic this fluid expulsion process, rendering them valuable components for microfluidic pumps in lab‐on‐a‐chip devices. CeFlowBot features an array of acoustically resonant bubbles strategically placed to replicate the pumping action observed in cephalopods, enhancing its propulsion capabilities. Furthermore, CeFlowBots are equipped with magnetic layers, allowing them to be steered through a synergistic combination of magnetic and acoustic fields. Notably, the modulation of acoustic power in CeFlowBots enables them to grasp and release nearby objects in their environment. This remarkable ability to navigate remote environments under the influence of magnetoacoustic fields and execute precise manipulations positions CeFlowBots as a valuable asset in clinical applications, particularly in the realm of targeted drug delivery. To track their motion in challenging environments where optical cameras may be impractical, an ultrasound imaging system is employed to visualize the movements of the bots, offering a promising avenue for their deployment in otherwise hard‐to‐reach locations.

Moreover, Zhang et al.^[^ ^175^ ^]^ offer neutrobots, which are dual‐responsive (DR) hybrid neutrophil microrobots that can breach the blood–brain barrier (BBB) for active malignant glioma therapy in vivo. They can be magnetically activated to move intravascularly and exhibit chemotactic behavior along a gradient of inflammatory stimuli. The meticulous construction of the neutrobots occurs during the phagocytosis process, in which natural neutrophils take up magnetic nanogels that have been coated with drug‐loaded *Escherichia coli* membranes. Notably, the inclusion of *E. coli* membrane serves two functions: improving the effectiveness of phagocytosis and reducing drug leakage within the neutrophils, assuring accurate drug administration. These neutrobots exhibit autonomous aggregation in the brain and controllable intravascular mobility when subjected to a controlled rotating magnetic field. They also show an amazing capacity to cross the BBB, propelled by positive chemotactic motion along the gradient of inflammatory chemicals. In contrast to traditional drug injection techniques, this novel approach significantly reduces the multiplication of tumor cells using dual‐responsive neutrobots for targeted drug administration and is a showcase of the advancement of microrobots for biomedical applications in the brain.

## 9 Biomedical Applications of Micro/Nanorobots

The article explores into the recent advances in the realm of micro and nanorobots for biomedical uses. The actuation mechanisms of micro/nanorobots, in the context of their biomedical applications—including diagnosis, therapy, regenerative medicine, and surgery—are highlighted in **Table** **5**. Micro/nanorobots have the potential for deep tissue monitoring and performing intricate tasks. However, difficulties lie in controlling them in deep tissues, their fabrication, biocompatibility, and potential immune reactions.

**Table 5:** Biomedical applications of micro/nanorobots

Category	Actuation mechanism	Application	Example of organ/region	References
Diagnosis	Magnetic field	Photoacoustic, magnetic resonance, and fluorescent imaging	GI, human capillaries, tumour hypoxia, pathogen detection and sampling	[3, 64, 194, 195, 196, 197]
	Light driven ‐ Acoustic	Biosensor, Bacterial Infection Treatment	Medical analysis	[138, 198]
Therapeutic	External rotating magnetic field	Drug and gene delivery	Single‐cell and drug delivery, human embryonic kidney, and tumour regions	[199, 200]
	Magnetic (near‐infrared and pH‐responsive)	Drug and gene delivery, Cells transport	Liver, renal and breast cancer, human embryonic kidney, inflammation, and specific surgical sites	[54, 58, 66, 96, 187, 201, 202, 203, 204, 205, 206]
	Chemical actuation (pH responsive, enzyme‐powered, photoacoustic guided, chemotaxis)	Drug and gene delivery, molecular cargos delivery across living cells	Stomach, arthritis therapy, intestines, cancer immunotherapy, ischemic stroke inflammation, and renal cancer and human embryonic kidney	[201, 202, 203, 204, 206, 207, 208, 209, 210, 211, 212, 213, 214, 215]
	Biohybrid (Sperm, MTB l. Monocytogenes, Serratia marcescens, *Escherichia coli*, bacteria‐spirulina platensis, and macrophage)	Reproduction, targeted drug, gene and molecular cargos delivery across living cells	Oviduct, stomach, arthritis therapy, intestine, colon cancer, bladder tumour, carcinoma, breast, and cervical cancer	[152, 195, 199, 200, 201, 202, 203, 204, 205, 206, 207, 208, 209, 210, 211, 212, 213, 214, 215, 216, 217, 218, 219, 220, 221, 222, 223, 224, 225, 226, 227, 228, 229]
	Ultrasound	Drug delivery	colon carcinoma, cancer therapy	[123, 230]
Regenerative	Magnetic	Cell manipulation, cell‐encapsulating, and cells transport	Colon cancer, oviduct, cervical and renal cancer, myoblasts and tissue engineering	[43, 46, 56, 152, 199, 201, 202, 203, 204, 205, 216, 217, 218, 219, 220, 221, 222, 223, 224, 225, 226, 227, 228, 229, 231]
	Acoustic	Cell‐encapsulation and delivery	Tissue engineering (Cardiomyocyte)	[232]
	Phototactic acoustic	Tissue engineering	Explorative tissue	[233]

Although research in the field of micro/nanorobots is growing and still evolving, there is a need for intensive research to enhance their functionality and dependability. The eventual goal is to develop techniques to mass produce designs proven effective in vivo for practical usage. As this sector grows in terms of researchers and breadth of studies, the capabilities of these minuscule robots in biomedical realms become increasingly evident. Collaborative research could help surmount the present challenges, especially for clinical usage.

Micro/nanorobots show promise for procedures like targeted drug delivery, but there is still a gap before they can be clinically deployed. There is an emphasis on making micro/nanorobots harmless to the body after their tasks are completed, especially considering their need for removal post‐application. Most studies have not yet addressed their retrievability or decomposition, so creating fully biodegradable micro/nanorobots could be a leap forward. Commonly used magnetic materials for micro/nanorobots might be nontoxic, but some, like nickel, can harm body tissues. The absorbability of MNPs varies with their size. For instance, magnetic particles under 10 nm in size can be naturally expelled from the body through the kidneys in a matter of days. Meanwhile, particles bigger than 100 nm are slowly eliminated by the liver and spleen.

In the future, synthetic materials that are both biocompatible and biodegradable should be developed. Moreover, when micro‐organisms and cells are chosen for biohybrid micro/nanorobots, their safety within the human body must be ensured. Nonharmful micro‐organisms have been used by researchers to design these microrobots, but a method to prevent them from being detected by the immune system is often not included. Commensal bacteria, which are naturally found in our bodies and can avoid detection by the immune system due to their association with the host, can be utilized to construct biohybrid microrobots. Going forward, the use of commensal bacteria or micro‐organisms that possess cell‐mimicking properties might be considered an effective approach for the creation of biohybrid microrobots tailored for advanced localized therapy and tumour internalization. Additionally, the intricate immune responses to these biohybrid microrobots need to be understood beyond just the potential toxicity of the materials involved. On the topic of design inspiration, many biohybrid microrobots are influenced by micro‐organisms and cells. However, the current versions of these robots do not possess the responsive abilities found in natural organisms, like chemotaxis or thermotaxis. As an example, certain micro‐organisms react to external forces like magnetism or light, while sperm cells use various tactics to reach the egg. While modern biohybrid microrobots might match natural organisms in terms of movement, they have not fully harnessed the inherent capabilities of these organisms in medical settings. Truly mimicking these natural entities means not only copying their structures but also integrating their responsive behaviors, pushing the boundaries of biomimetic engineering.

Because the primary environment for these micro/nanorobots is the human body, they need the capability to function in complex situations and ensure sustained movement. Given the diverse conditions within the human body, it is essential for micro/nanorobots to adapt to the specific circumstances of individual organs or cells. For instance, microrobots used for GI delivery should resist stomach acid. When used in ocular medicine, these robots should navigate through the dense matrix in the vitreous. This penetrating ability is also vital for targeting tumors.

Various biological elements, like proteins, lipids, nucleic acids, and diverse pH levels, can affect micro/nanorobots motion. Their behavior in unique physiological fluids, which have intricate properties, is still not fully understood. Biological barriers, such as cell membranes and the BBB, also present challenges. In the field of targeted drug delivery, delivering therapeutic drugs to the cell requires overcoming the cell membrane barrier. Furthermore, micro/nanorobots can be identified and removed from the bloodstream by specific cells due to protein absorption on their surface. Ensuring long‐lasting, effective movement within the body is a significant hurdle for these robots.

Using swarms of nanorobots could enhance drug delivery efficiency. Clusters of nanorobots are also easier to monitor inside the body. Although tracking individual robots remains challenging due to imaging limitations, using swarms might lower this tracking challenge. The collective behavior of these swarms, influenced by various environmental factors, remains intricate and requires further study.

## 10 Future Research and Conclusion

The field of micro/nanorobotics has witnessed remarkable advancements in recent years, offering significant potential for applications in various medical domains. However, there are critical challenges that necessitate immediate attention and further investigation. Addressing these challenges and capitalizing on the benefits of micro/nanorobotics can revolutionize medical management, enabling less invasive procedures and targeted therapies in previously inaccessible anatomical locations. Here, we highlight key areas for future research and development:

### 10.1 Hybrid Actuation Modes

While individual actuation modes have been explored, there is a need to focus on hybrid actuation systems. Integrating multiple actuation modalities can leverage the strengths of each approach while mitigating their drawbacks. Hybrid actuation has the potential to enhance the performance, versatility, and adaptability of micro/nanorobots, enabling precise manipulation and control in various medical scenarios.

### 10.2 Motion Control Techniques

Beyond actuation, comprehensive investigations into motion control are essential.^[^ ^176^ ^]^ Real‐time monitoring and optimal control of micro/nanorobot operations require advancements in motion control techniques. Magnetic field control, ultrasonic control, photoelectric control, and chemical reaction control should be concurrently developed to enable precise and responsive navigation of micro/nanorobots within complex biological environments.

### 10.3 Manufacturing and Novel Materials

Ongoing advancements in nanotechnology present opportunities for novel materials and manufacturing techniques.^[^ ^177^ ^]^ Future research should focus on developing new production methods for micro/nanorobots while prioritizing the use of biocompatible nanomaterials. This approach can reduce costs and expand the applications of micro/nanorobots in biomedical fields, paving the way for personalized medicine and targeted therapies.

### 10.4 Clinical Testing and Usage

The clinical translation of micro/nanorobotics faces certain challenges that impede its progress. One key hindrance lies in the adoption of the necessary actuation technologies within medical settings. Regulatory barriers, effective control mechanisms, and the implementation of large‐scale systems in a safe and reliable manner are critical aspects that require attention. Ensuring the safety and efficacy of micro/nanorobots in clinical settings is of utmost importance. Thorough investigation of potential risks and benefits is necessary before their widespread adoption. Comprehensive examination of aspects such as biocompatibility, toxicity, and long‐term effects is crucial to install confidence in the medical community regarding the integration of micro/nanorobots into medical practice.

In conclusion, addressing the challenges and advancing research in these key areas will significantly contribute to the development of micro/nanorobots and their applications in medicine. The adoption of hybrid actuation modes and motion control techniques holds great promise for enabling less invasive procedures and targeted therapies. Concurrently, exploring novel materials and manufacturing techniques will expand the possibilities for micro/nanorobot applications. By thoroughly investigating the safety and efficacy of micro/nanorobots in clinical testing and usage, we can pave the way for transformative advancements in medical management, improving treatment efficacy and reducing post‐operative complications. The future of micro/nanorobotics in medicine is bright, offering immense potential for revolutionizing healthcare practices and positively impacting patient outcomes.

## Conflict of Interest

The authors declare no conflict of interest.
